# CEP-1, the *Caenorhabditis elegans* p53 Homolog, Mediates Opposing Longevity Outcomes in Mitochondrial Electron Transport Chain Mutants

**DOI:** 10.1371/journal.pgen.1004097

**Published:** 2014-02-27

**Authors:** Aiswarya Baruah, Hsinwen Chang, Mathew Hall, Jie Yuan, Sarah Gordon, Erik Johnson, Ludmila L. Shtessel, Callista Yee, Siegfried Hekimi, W. Brent Derry, Siu Sylvia Lee

**Affiliations:** 1 Department of Molecular Biology and Genetics, Cornell University, Ithaca, New York, United States of America; 2 Developmental and Stem Cell Biology, The Hospital for Sick Children, Toronto, Ontario, Canada; 3 Department of Biology, McGill University, Montréal, Quebec, Canada; Stanford University Medical Center, United States of America

## Abstract

*Caenorhabditis elegans* CEP-1 and its mammalian homolog p53 are critical for responding to diverse stress signals. In this study, we found that *cep-1* inactivation suppressed the prolonged lifespan of electron transport chain (ETC) mutants, such as *isp-1* and *nuo-6*, but rescued the shortened lifespan of other ETC mutants, such as *mev-1* and *gas-1*. We compared the CEP-1-regulated transcriptional profiles of the long-lived *isp-1* and the short-lived *mev-1* mutants and, to our surprise, found that CEP-1 regulated largely similar sets of target genes in the two mutants despite exerting opposing effects on their longevity. Further analyses identified a small subset of CEP-1-regulated genes that displayed distinct expression changes between the *isp-1* and *mev-1* mutants. Interestingly, this small group of differentially regulated genes are enriched for the “aging” Gene Ontology term, consistent with the hypothesis that they might be particularly important for mediating the distinct longevity effects of CEP-1 in *isp-1* and *mev-1* mutants. We further focused on one of these differentially regulated genes, *ftn-1*, which encodes ferritin in *C. elegans*, and demonstrated that it specifically contributed to the extended lifespan of *isp-1* mutant worms but did not affect the *mev-1* mutant lifespan. We propose that CEP-1 responds to different mitochondrial ETC stress by mounting distinct compensatory responses accordingly to modulate animal physiology and longevity. Our findings provide insights into how mammalian p53 might respond to distinct mitochondrial stressors to influence cellular and organismal responses.

## Author Summary

Perturbing different components of the mitochondrial electron transport chain (ETC) can affect the lifespan of *Caenorhabditis elegans* in divergent ways. ETC dysfunction can either attenuate or extend *C. elegans* lifespan. Here we demonstrate that the *C. elegans* homolog of mammalian p53, CEP-1, is key in mediating differential survival of distinct ETC mutants. Inactivating *cep-1* in two long-lived ETC mutants shortened their lifespans, whereas *cep-1* deletion in two short-lived ETC mutants restored their lifespans. Because how CEP-1 does this is unknown, we compared the genome-wide expression profiles of the long-lived *isp-1* and short-lived *mev-1* ETC mutants upon *cep-1* removal. We found that CEP-1 is able to differentially regulate a small subset of genes depending on the specific ETC mutations. We validated one of the genes, the iron transporter ferritin (*ftn-1*), to be an important target through which CEP-1 mediates distinct lifespan outcome in specific ETC mutants. We also identified numerous other candidate CEP-1 targets that merit future analysis, which will further elucidate how CEP-1 differentially responds to the distinct ETC dysfunction of various mitochondrial mutants. Our study provides new insights that will inform how mammalian p53, a critical tumor suppressor, might respond to mitochondrial and metabolic stresses.

## Introduction

Mitochondria are major sites of numerous metabolic processes, in particular electron transport and ATP production, and are essential for life. Not surprisingly, mitochondria also play central roles in aging and disease [1]. In model organisms such as worms, flies, and mice, specific point mutations or RNAi knockdowns directly affecting the electron transport chain (ETC) result in varying effects on development and longevity, ranging from developmental arrest and shortened survival to extended lifespan. The extended lifespan associated with moderate mitochondrial ETC dysfunction was surprising and further highlights the complex relationship between mitochondrial function and aging. An emerging model posits that a moderate reduction in mitochondrial ETC function can lead to compensatory responses that lengthen lifespan [2]–[5], whereas a more severe reduction in mitochondrial ETC function, beyond an innate threshold, will lead to developmental arrest and/or early death [6]–[7]. How different degrees of mitochondrial dysfunction result in opposing effects on longevity remains largely unknown.

*Caenorhabditis elegans* represents a powerful model to study the genetic basis of cellular and organismal changes in response to mitochondrial dysfunction. Previous findings in *C. elegans* have revealed a number of long-lived and short-lived ETC mutants. The *nuo-6(qm200)* mutant, which harbors a point mutation in the NADH-ubiquinone oxidoreductase of complex I, the *isp-1(qm150)* mutant, which harbors a point mutation in the rieske iron sulphur subunit of complex III, and the *clk-1(e2519)* mutant, with a point mutation in a coenzyme Q biosynthesis enzyme, exhibit substantial lifespan extension [8]–[9]. In contrast, the *mev-1(kn-1)* mutant, with a point mutation in the succinate dehydrogenase subunit c of complex II, and the *gas-1(fc21)* mutant, with a point mutation in the NADH:ubiquinone oxidoreductase NDUFS2 subunit of complex I, live significantly shorter than wild-type worms [10]. Furthermore, large-scale RNAi screens have revealed that RNAi-mediated inactivation of many of the ETC subunits result in prolonged or shortened lifespan [11].

Studies using genetic mutants and RNAi-mediated knockdown of ETC components in worms have begun to reveal the mechanistic basis of the longevity outcomes associated with mitochondrial dysfunction. Reactive oxygen species (ROS) have emerged as an important signaling intermediate in the ETC mutants. Specifically, *nuo-6*, *isp-1*, and *mev-1* mutants have been shown to exhibit elevated levels of mitochondrial superoxide, and antioxidant treatment of these worms was able to revert their longevity phenotype [12]–[13]. Interestingly, the long-lived *clk-1* mutant was not found to exhibit a higher level of mitochondrial superoxide, and antioxidant treatment had no impact on its lifespan, suggesting that the *clk-1* mutation influences lifespan independent of ROS. In addition to increased ROS levels, the ETC mutants also exhibit an altered metabolism. The long-lived *nuo-6*, *isp-1*, and *clk-1* mutants share similar metabolic profiles that are distinct from that of the *mev-1* short-lived mutant [14]–[15]. An elevated production of metabolites, such as α-ketoacids and α-hydroxyacids, has been proposed to act as a pro-longevity signal in the long-lived ETC mutants. Furthermore, studies that have largely employed RNAi-mediated knockdown of various ETC subunits demonstrated that an imbalanced stoichiometry of the ETC protein subunits triggered a strong mitochondrial unfolded protein response (mtUPR). In this scenario, processed peptides in the mitochondria are thought to serve as the signal that activates several transcriptional regulators, including UBL-5 and ATFS-1, to induce transcriptional responses necessary to restore proteostasis in the mitochondria, which contributes to longevity determination [16]. Lastly, several RNAi and candidate screens have identified additional transcription factors, such as CEP-1 [17], CEH-23 [18], and TAF-4 [19] that mediate the lifespan of various ETC mutants.

The transcription factor p53 has recently emerged as a key regulator of metabolic balance [20]–[22]. Despite its importance, how p53 senses metabolic stress and accordingly regulates molecular changes that determine the physiological outcomes of an organism remains poorly understood. *C. elegans* CEP-1, the sole homolog of the mammalian p53 family [23] (p53, p63 and p73), is known to mediate the lifespan changes in worms with mitochondrial dysfunction. Inactivation of *cep-1* has been shown to partially suppress the extended longevity of *isp-1* mutant worms [24]. Furthermore, using different concentrations of RNAi to cause different degrees of knockdown of several ETC components demonstrated that CEP-1 is required for the increased longevity under mild mitochondrial disruption as well as the shortened lifespan when mitochondrial damage is more severe [25]. Therefore, CEP-1 exerts opposite effects on lifespan that likely depend on the levels of mitochondrial stress experienced. The underlying mechanism governing this intriguing duality of CEP-1 function is not known.

In this study, we sought to further characterize the role of CEP-1 in the longevity of several mitochondrial ETC mutants. Our results indicate that CEP-1 is a critical mediator of the lifespan of several mitochondrial mutants, suggesting that CEP-1 plays a central role in sensing mitochondrial distress and coordinating physiological outcomes accordingly. We also evaluated the CEP-1-regulated transcriptomes in the long-lived *isp-1* and the short-lived *mev-1* mitochondrial ETC mutants. Despite the opposing roles that CEP-1 appears to play in determining the lifespan of these mutants, the CEP-1-regulated transcriptional profiles were largely similar in these mutants. Nevertheless, the expression of a small group of genes was differentially regulated by CEP-1 between the long-lived *isp-1* and the short-lived *mev-1* mutants. Interestingly, this small group of genes is enriched for the Gene Ontology functional group “aging”, indicating they are over-represented by genes previously known to have a role in aging in worms. We functionally validated one of these differentially regulated genes, *ftn-1*, which encodes ferritin in *C. elegans*, by demonstrating that its RNAi-mediated depletion significantly impacted the lifespan of the *isp-1* but not *mev-1* mutant. This result supports our hypothesis that CEP-1 can differentially regulate a small subset of target genes to achieve distinct longevity outcomes in response to mutations in different ETC components.

## Results

### CEP-1 exerts opposing effects on longevity and development in the long-lived *isp-1* and short-lived *mev-1* mutants

**Figure 1:**
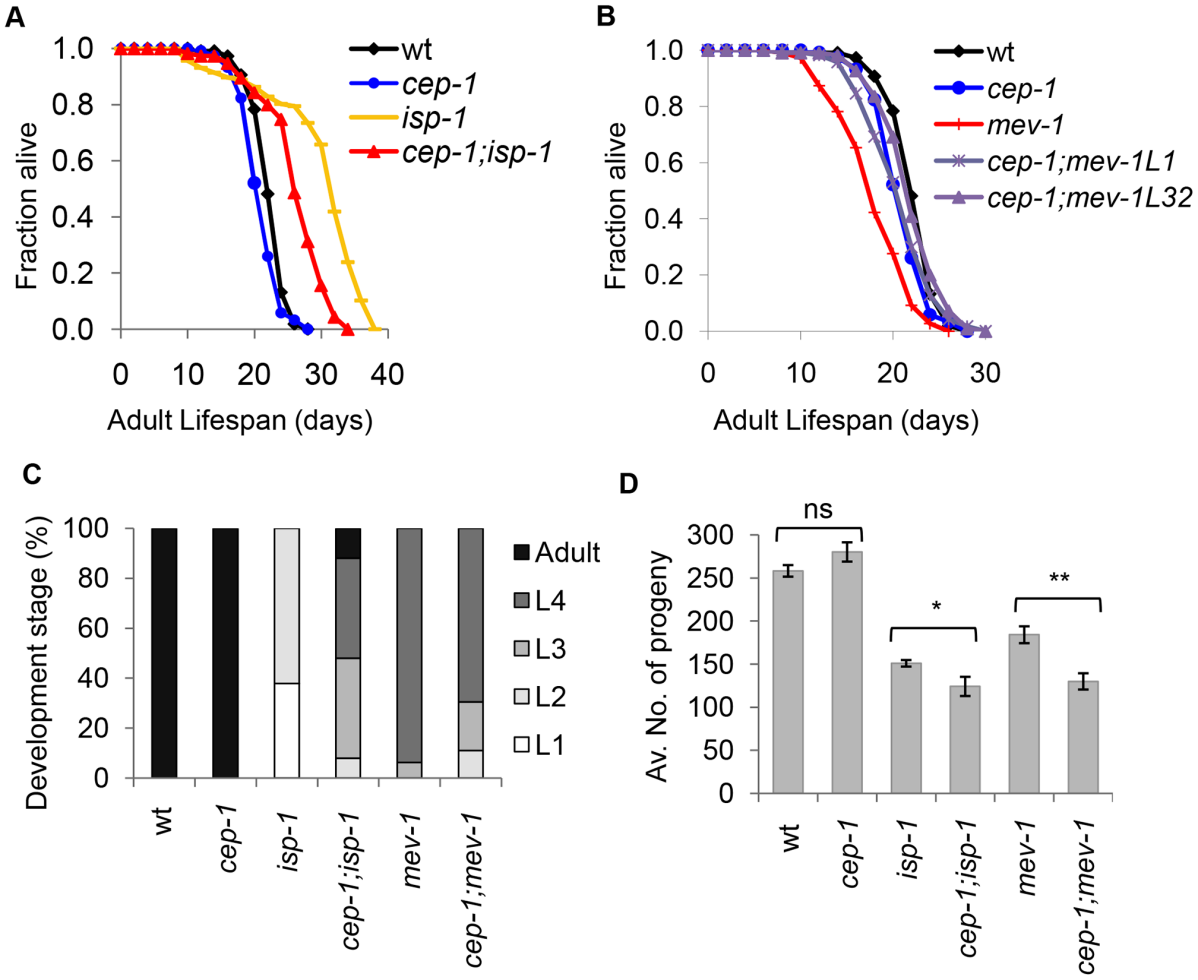
CEP-1 mediates the longevity and development of two mitochondrial mutants in *C. elegans*. (A) *cep-1* mutation partially suppresses *isp-1* mutant longevity as the *cep-1;isp-1* double mutant lifespan is shorter than that of the *isp-1* single mutant. (B) *cep-1* mutation restores the *mev-1* mutant lifespan as the lifespans of two *cep-1;mev-1* isolates (L1, L32) are similar to that of wt. (C) The percentage of worms at each developmental stage was quantified for wt, *cep-1, isp-1, cep-1;isp-1, mev-1*, and *cep-1;mev-1* mutant worms after 60 hr of growth from the embryonic stage at 20°C. (D) The average number of progeny production for each line was calculated from 5 to 10 worms. The *isp-1* and *mev-1* mutants produce significantly less progeny than wt. The *cep-1;isp-1* and *cep-1;mev-1* double mutants display significantly lower brood sizes than their respective single mutant controls (*p<0.05, **p<0.0005). The error bars represent standard errors. Statistical analysis was performed using a two-tailed t-test.

Previous results suggested that *cep-1* is required for the lifespan extension associated with mild mitochondrial dysfunction and the shortened lifespan associated with severe mitochondrial dysfunction [25]. We confirmed these results and demonstrated that inactivation of *cep-1* partially but consistently suppressed the extended lifespan of *isp-1* mutant animals (Fig. 1A), (Fig. S1A, Table S1, S2). We further showed that inactivation of *cep-1* largely restored the lifespan of *mev-1* mutants to wild-type (Fig. 1B), (Fig. S1A, Table S1, S2). Our data are therefore consistent with previous findings suggesting that CEP-1 can respond to different degrees of mitochondrial dysfunction and modulate lifespan accordingly.

In addition to lifespan changes, mitochondrial ETC mutants also develop slowly and display reduced brood sizes. To assess whether CEP-1 participates in the development of mitochondrial ETC mutants, the development time of *isp-1* and *mev-1* mutants with or without *cep-1* was compared to that of wild-type (wt) worms. Synchronized embryos of the various strains were allowed to develop at 20°C for 60 hours, and the number of adults and larvae were counted (Fig. 1C, Table S3). The data showed that wt and *cep-1* mutant worms developed at similar rates, and 100% of the populations had reached adulthood by 60 hr. As expected, the *isp-1* mutant worms grew slowly, and the majority were in the L1 (37%) and L2 (62%) stages after 60 hr. However, *cep-1;isp-1* double mutants developed noticeably faster, and the majority were L3s (40%), L4s (40%), and adults (12%) after 60 hr. These data suggest that *cep-1* inactivation partially recues the slow development of the *isp-1* mutant. Interestingly, *cep-1* inactivation exerted an opposite effect on *mev-1* mutant development. The *mev-1* mutants were slightly developmentally delayed, where the majority of *mev-1* mutant worms were in the L3 (6%) and L4 (93%) stages at 60 hr. The development rate of the *cep-1;mev-1* double mutant worms was heterogeneous and further delayed (L2 (11%), L3 (19%) and L4 (69%) at 60 hr) compared to *mev-1* single mutant worms.

To examine a possible role for CEP-1 in the reproduction of mitochondrial ETC mutants, the average brood size of *isp-1* and *mev-1* mutants with or without *cep-1* was compared to that of wt animals. The brood size of the *cep-1* mutant did not significantly differ from wt (*p* = 0.1), and, as expected, the mitochondrial mutants (*isp-1, mev-1*) displayed significantly lower brood sizes compared to wt (*p*<0.0001). The double mutants *cep-1;isp-1* and *cep-1;mev-1* exhibited a further brood size reduction compared to *isp-1* and *mev-1* single mutants, respectively (*p*≤0.05) (Fig. 1D, ). These results suggest that *cep-1* inactivation further exacerbates the reproductive defect associated with mitochondrial dysfunction.

Taken together, *cep-1* appears to participate in multiple physiological outcomes of ETC mutants. *cep-1* activity promotes, at least partially, both the slower development and the longer lifespan of the *isp-1* mutant but is required to prevent further reproductive deterioration. In contrast, *cep-1* activity promotes a shortened *mev-1* mutant lifespan but is required for a relatively normal developmental rate. Similar to its role in *isp-1* reproduction, *cep-1* prevents reproductive decline in *mev-1* mutants. Since *cep-1* loss similarly impacts the reproductive success of *isp-1* and *mev-1* mutants, but its inactivation has opposing effects on the lifespan and developmental rate of these mutants, the role of *cep-1* in reproduction might be independent of its role in lifespan and development in ETC mutants.

### CEP-1 mediates reduced physiological germline apoptosis in the long-lived *isp-1* mutant

**Figure 2:**
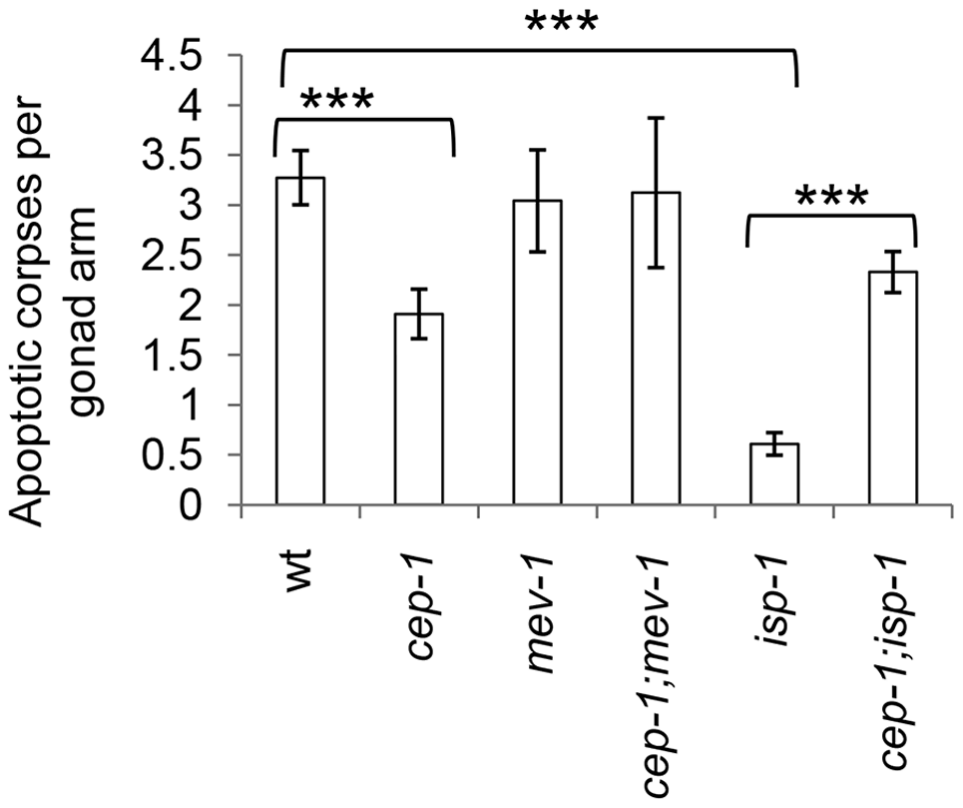
CEP-1 mediates reduced physiological germline apoptosis in the *isp-1* mutant. Physiological levels of apoptosis were quantified by counting the number of apoptotic corpses per gonad arm in various *C. elegans* strains. The corpses were counted using DIC microscopy at 63× magnification 48 hr post L4. The data represent the average of at least 3 independent experimental replicates (n≥15 gonad arms for each) ± standard error. Statistical analysis was done using the Mann-Whitney U-test. ***p<0.001.

CEP-1 is a well-established key regulator of stress-induced apoptosis [24]. Since CEP-1 is a crucial mediator of the longevity outcomes of *isp-1* and *mev-1* mutants, we asked whether CEP-1 does so by modulating apoptosis in these mutants. While apoptosis occurs throughout embryonic and larval development in *C. elegans*, we reasoned that monitoring apoptosis in adults would be more relevant in investigating adult lifespan. In *C. elegans* adults, physiological and stress-induced apoptosis occurs in the germline. We monitored physiological apoptosis in the germline of wild-type, *isp-1* and *mev-1* mutants with or without *cep-1* inactivation. CEP-1 is best characterized for its role in stress-induced apoptosis, and our data indicated that loss of *cep-1* alone only mildly affected physiological apoptosis in the germline. The short-lived *mev-1* mutant and *mev-1;cep-1* double mutant exhibited wt levels of physiological germline apoptosis (Fig. 2). Interestingly, the long-lived *isp-1* mutant displayed a significantly lower level of physiological germline apoptosis, which was completely rescued in the *cep-1;isp-1* double mutant (Fig. 2). These data suggest that CEP-1 protects against physiological germline apoptosis in the *isp-1* mutant. As CEP-1 has largely been demonstrated to promote apoptosis in *C. elegans*, this new role of CEP-1 in protecting against apoptosis merits further investigation.

### CEP-1-mediated transcriptional profiles in long-lived *isp-1* and short-lived *mev-1* mutants

CEP-1 appears to dually mediate the lifespan of mitochondrial mutants. We hypothesized that moderate mitochondrial dysfunction initiates a CEP-1-dependent defensive response that promotes longevity in long-lived mitochondrial mutants. Conversely, decreasing mitochondrial function beyond a threshold in short-lived mitochondrial mutants likely engages CEP-1 in a different way that results in a shortened lifespan. Since mammalian p53 is a well-established transcription factor, we sought to investigate the transcriptional response induced by CEP-1 in both a long-lived, *isp-1*, and a short-lived, *mev-1*, mitochondrial mutant. We hypothesized that the genes differentially regulated by CEP-1 between *isp-1* and *mev-1* mutants may mediate the distinct CEP-1 lifespans of the *isp-1* and *mev-1* mutants (see below). We compared the transcriptional profiles of synchronized *isp-1(qm150)* and *cep-1(gk138);isp-1(qm150)* young adults, as well as *mev-1(kn1)* and *cep-1(gk138);mev-1(kn1)*, using the Agilent 4×44K oligonucleotide microarray (Table S4).

**Figure 3:**
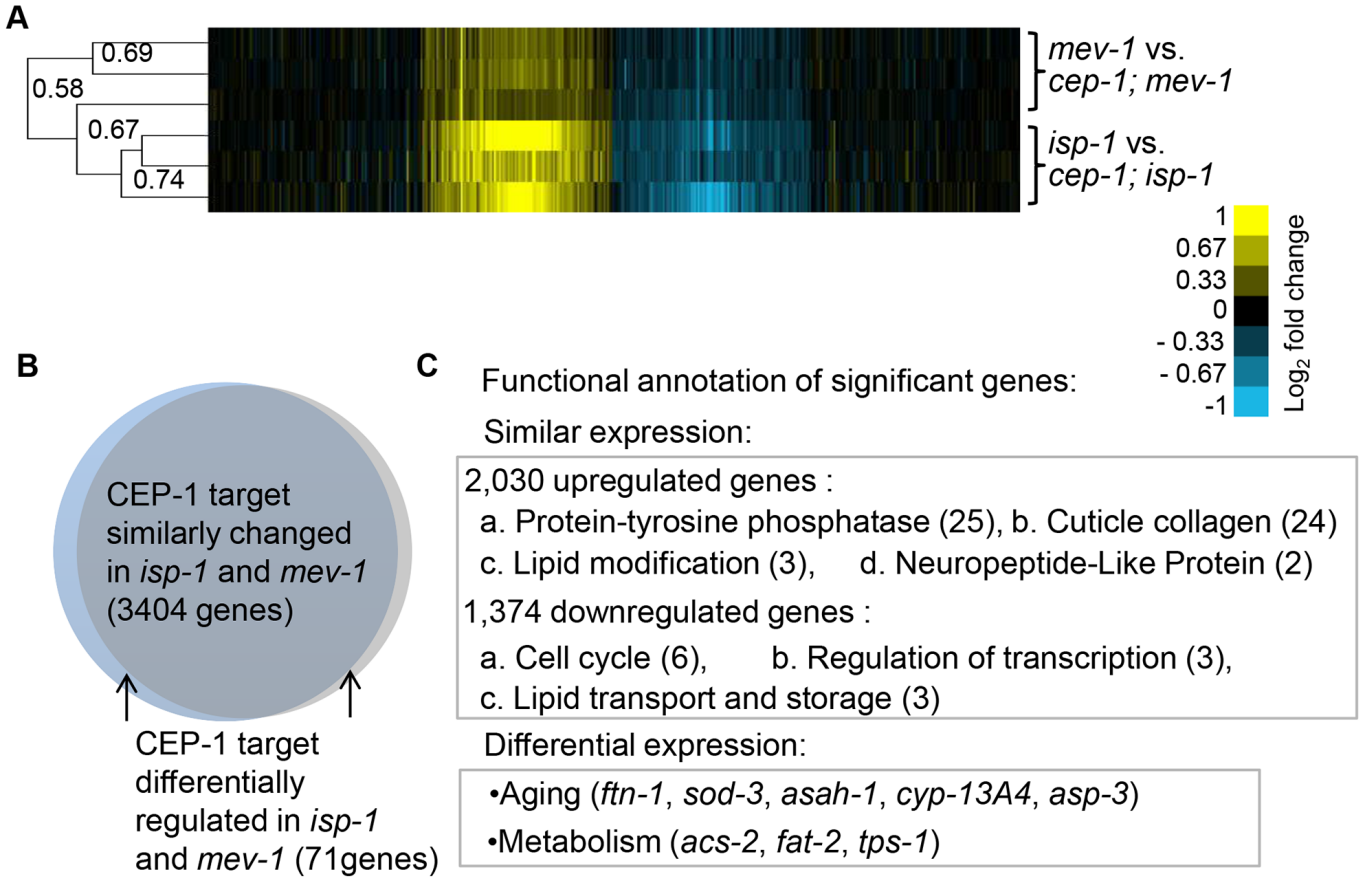
CEP-1-regulated transcriptomes in *isp-1* and *mev-1* mutants. (A) CEP-1-regulated genes in *isp-1* and *mev-1* mutants are largely similar. Hierarchical single linkage gene clustering was performed, and the dendrogram shows the clustered relationship of individual arrays. The numbers on the dendrogram represent the correlation coefficients between arrays. Yellow: upregulated, Blue: downregulated, Black: no change. (B) The expression of CEP-1-regulated genes that were significantly changed in *isp-1* and *mev-1* mutants, identified by SAM analysis, are represented in the Venn diagram. (C) DAVID functional annotation of similarly and differentially expressed CEP-1-regulated genes in *isp-1* and *mev-1* mutants. The numbers represent the enrichment scores for each group (score>1.3 is considered as significant). Several examples of aging and metabolic genes are listed.

Although CEP-1 exerts opposing effects on the lifespans of *isp-1* and *mev-1* mutants, hierarchical gene cluster analysis revealed that the CEP-1-regulated transcriptional profiles in long-lived *isp-1* and short-lived *mev-1* mutants were largely similar (correlation coefficient of 0.58) (Fig. 3A). Only a small number of genes exhibited *cep-1*-dependent differential regulation. Data analysis using the statistical tool SAM (Significance Analysis of Microarray) with a FDR (false discovery rate) of 0.5% revealed that CEP-1 regulated the expression of 3,404 genes (Table S5) in a similar manner in the long-lived *isp-1(qm150)* and short-lived *mev-1(kn1)* mutants (Fig. 3B, Fig. S2A). SAM analysis with a FDR of 1% followed by a gene list comparison also revealed 71 genes (Table S6, see Materials & Methods for details on gene list filtering) that were differentially regulated by CEP-1 in the *isp-1(qm150)* and *mev-1(kn1)* mutants (Fig. 3B, Fig. S2B). The expression of these genes largely differed quantitatively rather than qualitatively, i.e., they generally showed a greater CEP-1-mediated regulation in the *isp-1* or *mev-1* mutant backgrounds. To validate our microarray findings, we selected six genes (*F15E6.8/dct-7*, *C37A5.2/fipr-22*, *F28C6.1/AP-2 like TF*, *C52D10.6/skr-12, C08A9.1/sod-3 and F57B9.9/abu-13)* based on their classification by SAM analysis and performed quantitative reverse transcription PCR (qRT-PCR) using an independent biological sample. The expression changes of all six genes echoed the microarray results (Fig. S3).

To examine the biological processes of the CEP-1-regulated genes identified by SAM, we performed Gene Ontology (GO) analysis using DAVID (Database for Annotation, Visualization, and Integrated Discovery). We focused on the GO term categories that were most significantly enriched in our dataset compared to the distribution in the *C. elegans* genome. Interestingly, the small group of CEP-1-regulated genes that differentially changed in *isp-1* and *mev-1* mutants were enriched for genes with known roles in aging and metabolism (Fig. 3C, Table S7C). This finding supports our hypothesis that the genes differentially regulated by CEP-1 in *isp-1* and *mev-1* mutants are likely particularly important for the opposing effects on lifespan that CEP-1 exerts in these mutants. The genes that were similarly regulated by CEP-1 in *isp-1* and *mev-1* mutants likely account for the effects CEP-1 has on development, reproduction, and other physiological changes in these mutants and might also contribute to the final longevity (Fig. 3C, Tables S7A, S7B).

### The CEP-1-regulated gene *ftn-1* contributes to the longer lifespan of the *isp-1* mutant

**Figure 4:**
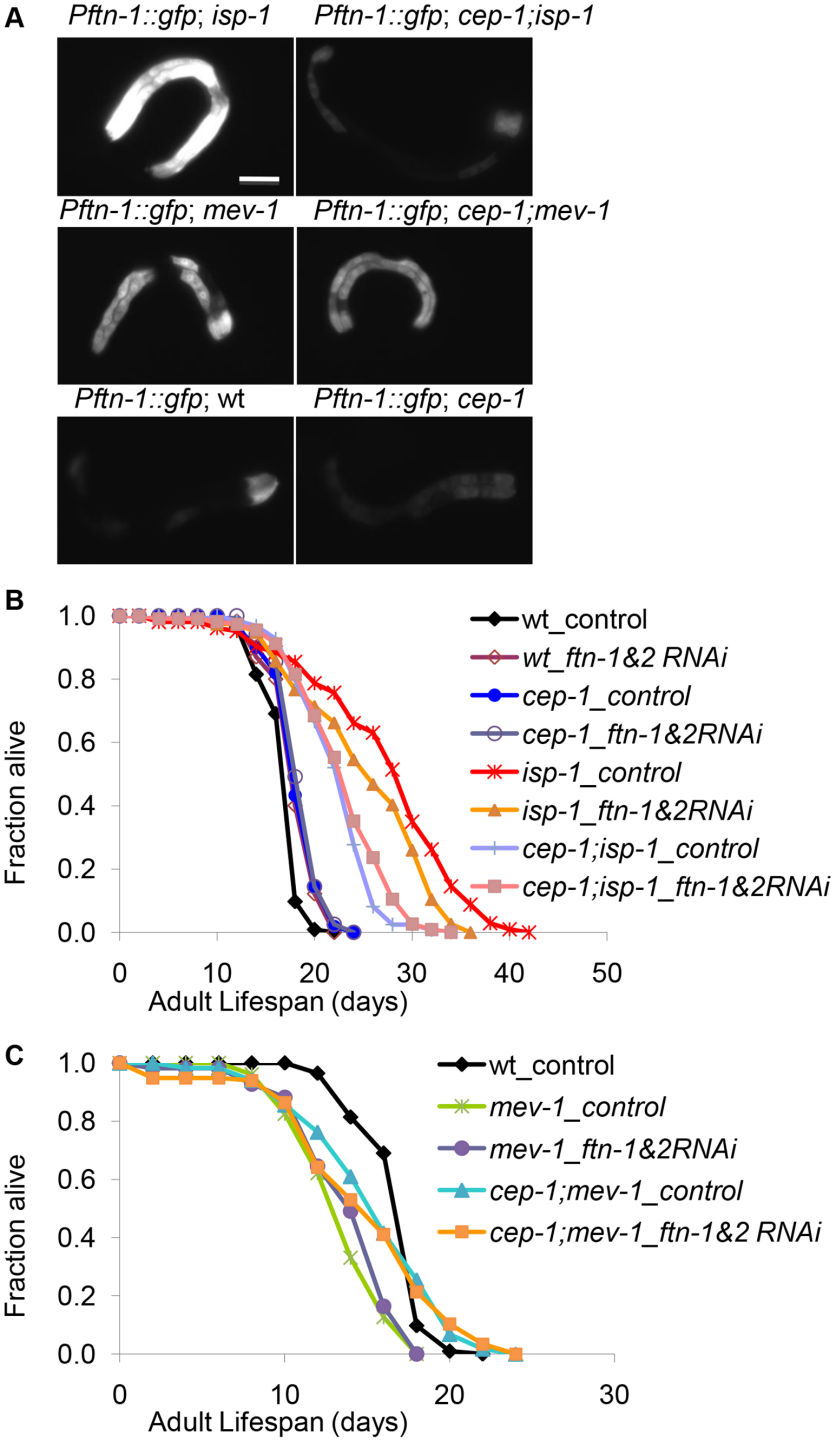
CEP-1-regulated ferritin induction partially mediates the extended lifespan of *isp-1* mutants. (A) *Pftn-1::gfp* expression in wt, *cep-1, isp-1, cep-1;isp-1, mev-1*, and *cep-1;mev-1* mutant worms. Scale bar = 100 µm. (B, C) The lifespans of wt, *cep-1, isp-1, cep-1;isp-1, mev-1* and *cep-1;mev-1* mutant worms treated with *ftn-1* and *ftn*-2 double RNAi. L4440 is a treatment control.

The microarray results helped narrow our analysis to a small number of CEP-1-regulated genes (Fig. 3C) that might contribute to the dual effects CEP-1 exerts on the longevity of mitochondrial mutants with varying degrees of dysfunction. One of these genes, *ftn-1*, encodes the *C. elegans* homolog of the ferritin heavy chain. Using qRT-PCR, we observed that *ftn-1* expression was repressed ∼2 fold in the *cep-1* mutant and induced ∼1.5 fold in the *isp-1* mutant compared to wt. The *cep-1;isp-1* double mutant displayed a similar repressed level of *ftn-1* expression as observed for the *cep-1* single mutant (Fig. S4A). While *ftn-1* expression was induced in the *mev-1* mutant, similar to the *isp-1* mutant, this induction was not changed in the *cep-1;mev-1* double mutant (), consistent with the microarray results. Inspection of the *ftn-1* upstream sequence did not reveal a known p53 binding motif, suggesting that CEP-1 might not directly regulate *ftn-1* transcription. *C. elegans* harbors another *ftn-1* homolog, *ftn-2*, but its expression was not changed in the ETC mutants compared to wt (Fig. S4B). Lastly, we used the *Pftn-1::gfp* strain, where a GFP reporter is fused to the *ftn-1* promoter [26], to assess whether the microarray and qRT-PCR results translated into observably meaningful *ftn-1* expression changes. *Pftn-1::gfp* was substantially induced in *isp-1* mutants and was completely suppressed in *cep-1;isp-1* double mutants (Fig. 4A), consistent with our microarray and qRT-PCR results. Additionally, *Pftn-1::gfp* expression was slightly induced in *mev-1* mutants and unchanged in *cep-1;mev-1* double mutants. Taken together, our data indicate that CEP-1 is important for ferritin regulation in wt and *isp-1* mutants but is dispensable in the *mev-1* mutant.

Ferritin regulates the storage and release of iron. As iron homeostasis is known to play an important role during mitochondrial dysfunction, we wanted to investigate whether ferritin regulation was responsible for the dual roles of CEP-1 in mitochondrial ETC mutant longevity. To assess whether ferritin upregulation promoted *isp-1* mutant longevity, we examined the lifespan of *isp-1* and *cep-1;isp-1* mutants after *ftn-1* and *ftn-2* knockdown by RNAi. We knocked down *ftn-1* and *ftn-2* simultaneously to prevent possible functional redundancy, which may preclude observable phenotypes when either gene is knocked down alone. Double RNAi-mediated knockdown of *ftn-1* and *ftn-2* significantly suppressed the extended lifespan of *isp-1* mutants, although not to the same degree as *cep-1* inactivation. Importantly, *ftn-1/2* RNAi did not further suppress the lifespan of the *cep-1;isp-1* double mutant (Fig. 4B, Fig. S5), suggesting that *cep-1* and *ftn-1/2* act in the same genetic pathway to mediate *isp-1* longevity, corroborating our model that *ftn-1/2* are downstream targets of CEP-1. Consistent with our expression results suggesting that *ftn-1/2* are not regulated by CEP-1 in the short-lived *mev-1* mutant, the *mev-1* mutant lifespan was not affected by *ftn-1/2* RNAi (Fig. 4C, Fig. S5).

### CEP-1 is key to physiological changes in multiple ETC mutants

**Figure 5:**
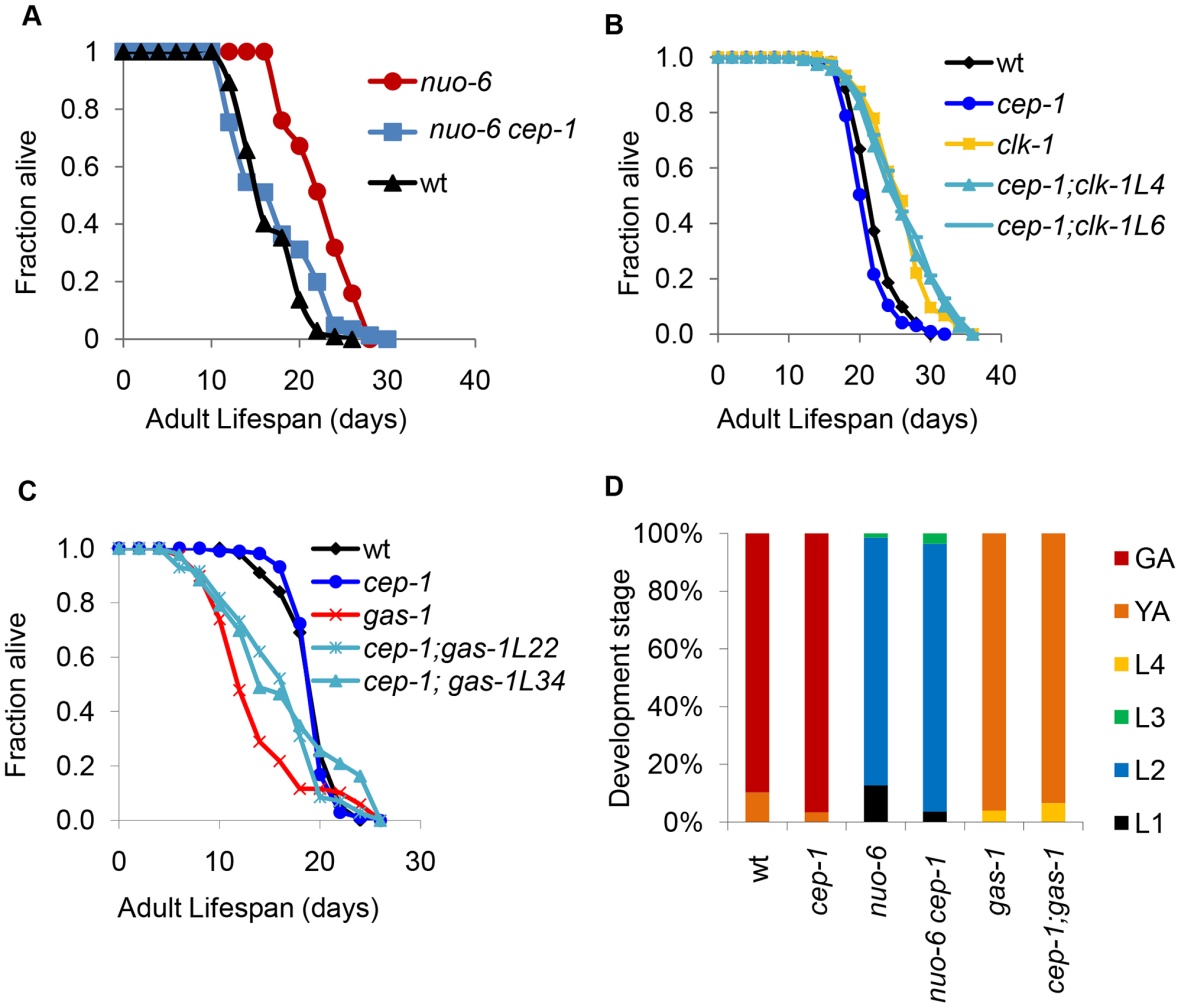
CEP-1 mediates the longevity and development of several mitochondrial mutants in *C. elegans*. (A) *cep-1* mutation fully suppresses the long lifespan of the *nuo-6* mutant. (B) *cep-1* mutation does not suppress *clk-1* mutant longevity as the lifespans of two *cep-1;clk-1* double mutant isolates (L4, L6) are similar to that of the *clk-1* single mutant. (C) *cep-1* mutation partially restores *gas-1* mutant lifespan as two isolates of *cep-1;gas-1* (L22, L34) live longer than the *gas-1* single mutant. (D) The percentage of worms at each developmental stage was quantified as described in Fig. 1C. *cep-1* deletion has little impact on *nuo-6* and *gas-1* mutant development.

Given the important role CEP-1 plays in determining the lifespan of *isp-1* and *mev-1* mutants, we next tested whether *cep-1* is generally required for the longevity of additional ETC mutants that have been well characterized in *C. elegans*. We explored the long-lived *nuo-6(qm200)* mutant, which harbors a point mutation in the NADH-ubiquinone oxidoreductase of complex I, the long-lived *clk-1(e2519)* mutant, which harbors a point mutation in a coenzyme Q biosynthesis enzyme, and the short-lived *gas-1(fc21)* mutant, which has a point mutation in the NADH:ubiquinone oxidoreductase NDUFS2 subunit of complex I. The *cep-1* null mutation largely suppressed the long-lived phenotype of the *nuo-6* mutant but did not affect the longevity of the *clk-1* mutant, as the *cep-1;clk-1* double mutant lived as long as the *clk-1* single mutant (Fig. 5A, 5B, Fig. S1A). Furthermore, *cep-1* deletion rescued the short-lived phenotype of the *gas-1* mutant, similar to its effect on the *mev-1* mutant lifespan (Fig. 5C, Fig. S1A). Taken together, the data suggest that CEP-1 crucially participates in the longevity outcome of multiple ETC mutants.

The fact that *cep-1* abrogation did not affect the *clk-1* mutant lifespan was intriguing. All of the well-characterized mitochondrial ETC mutants in *C. elegans* (*isp-1*, *nuo-6*, *mev-1*, and *gas-1*), with the exception of *clk-1*, have been shown to harbor elevated levels of mitochondrial superoxide. In fact, antioxidant treatment of all of these mutants, again with the exception of *clk-1*, has been shown to suppress (the long-lived) *isp-1* and *nuo-6* or rescue (the short-lived) *mev-1* and *gas-1* mutant lifespans. Therefore, the *cep-1*-mediated lifespans of these mitochondrial ETC mutants parallel the proposed roles that elevated ROS is exerting in these mutants, i.e., CEP-1 mediates the lifespans of mutants with elevated ROS but not of mutants without. This observation suggests the interesting possibility that CEP-1 might somehow be linked to the ROS-mediated longevity increases observed in the long-lived mitochondrial ETC mutants. Future studies aimed at thoroughly investigating the relationship between CEP-1 and ROS-mediated longevity will likely yield fruitful insights.

As *cep-1* impacted the development rates of *isp-1* and *mev-1* mutants, we tested whether *cep-1* similarly affected the development of *nuo-6* and *gas-1* mutants. Our data indicated that *cep-1* deletion had little impact on *nuo-6* and *gas-1* mutant development (Fig. 5D), unlike what we found for *isp-1* and *mev-1* mutants (Fig. 1C). Therefore, whereas *cep-1* mediates the lifespans of all four ETC mutants tested here (*isp-1*, *nuo-6*, *mev-1*, and *gas-1*), its involvement in the development of each of the mutants is mixed, suggesting that the role of CEP-1 in development may be distinct from its role in longevity in these various mutants.

**Figure 6:**
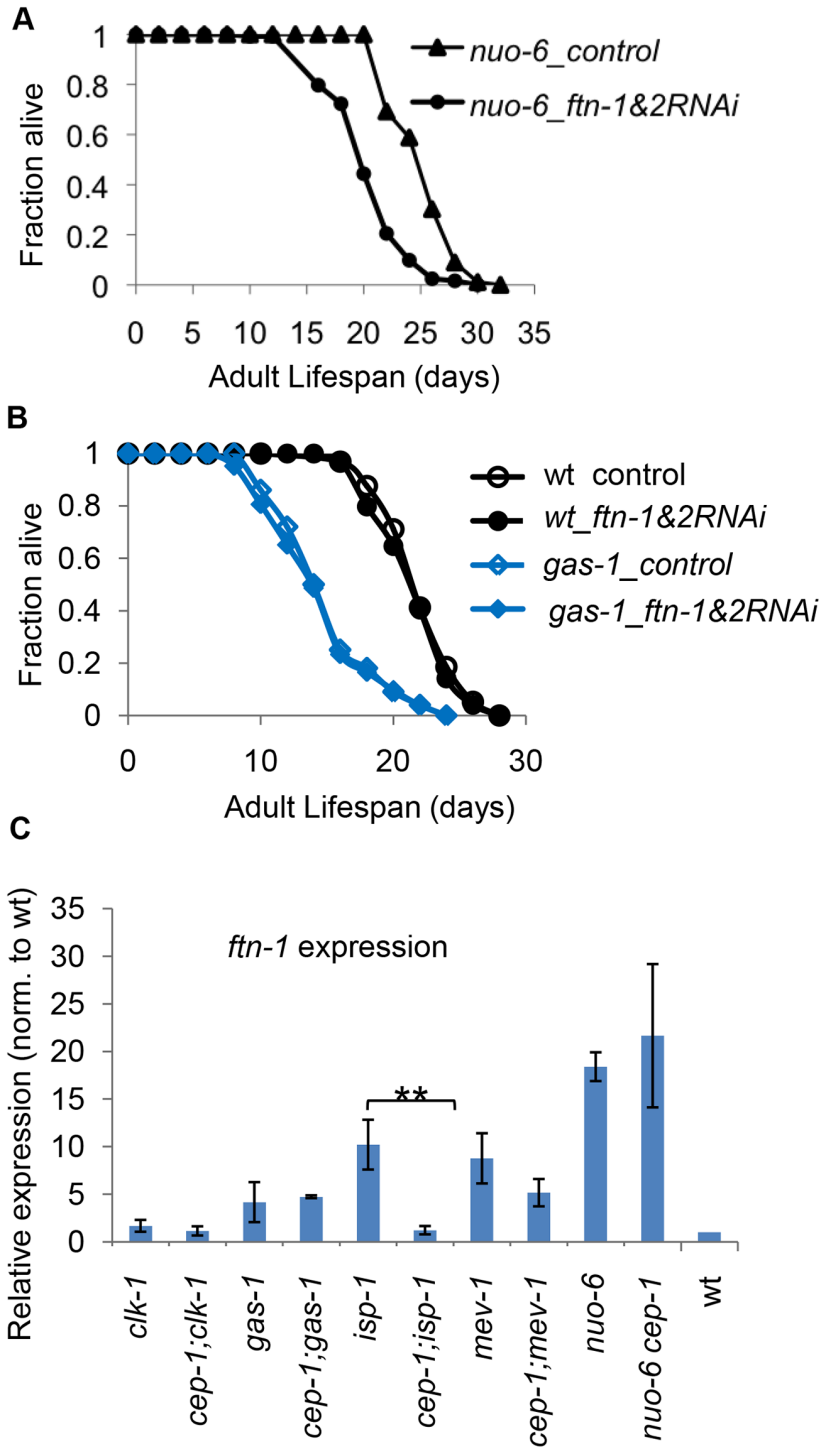
*ftn-1* is differentially expressed in various ETC mutants and mediates their lifespan outcomes. (A, B) RNAi-mediated knockdown of *ftn-1* attenuates the long life of the *nuo-6* mutant but does not impact the lifespan of the short-lived *gas-1* mutant. (C) qRT-PCR results of *ftn-1* expression levels in various ETC mutants. The two-tailed student t-tests were performed to determine significant difference in *ftn-1* expression levels with and without CEP-1 in each ETC mutant background.

Given that we observed an important role for CEP-1 in *ftn-1* regulation and iron homeostasis in ETC mutant longevity, we further tested whether *ftn-1* & *ftn-2* are regulated by CEP-1 in *nuo-6* and *gas-1* mutants and whether *ftn-1/2* are required for their longevity. Strikingly, *ftn-1* was greatly induced in the *nuo-6* mutant, even more so than in the *isp-1* mutant (Fig. 6C). Somewhat surprisingly, *cep-1* was not required for this induction, unlike in the *isp-1* mutant. We also demonstrated that RNAi-mediated depletion of *ftn-1/2* substantially suppressed the extended lifespan of the *nuo-6* mutant, similar to their depletion in the *isp-1* mutant (Fig. 6A). Although CEP-1 is essential for *ftn-1* induction in the *isp-1* mutant, it is dispensable in the *nuo-6* mutant; however, both *cep-1* and *ftn-1/2* are important for the longevity of *nuo-6* and *isp-1* mutants. Therefore, while iron homeostasis and *cep-1* are both important for longevity determination in the long-lived *isp-1* and *nuo-6* mutants, CEP-1 regulates *ftn-1* only in the *isp-1* mutant. We hypothesize that another transcription factor likely regulates *ftn-1* expression in the *nuo-6* mutant. Interestingly, *ftn-1* was induced in the short-lived *gas-1* mutant independently of *cep-1*, similar to that in the short-lived *mev-1* mutant (Fig. 6C). Additionally, depleting *ftn-1/2* did not rescue the shortened lifespan of the *gas-1* mutant, similar to that observed in the *mev-1* mutant (Fig. 6B). Therefore, iron homeostasis might be a determinant of *isp-1* and *nuo-6* longevity, but it does not appear to be important for mediating the lifespans of *gas-1* and *mev-1* mutants. Lastly, *ftn-1* was not induced in the *clk-1* mutant, another indication that *clk-1* mutant might engage a different mechanism to extend lifespan compared to the other mitochondrial ETC mutants studied here.

**Figure 7:**
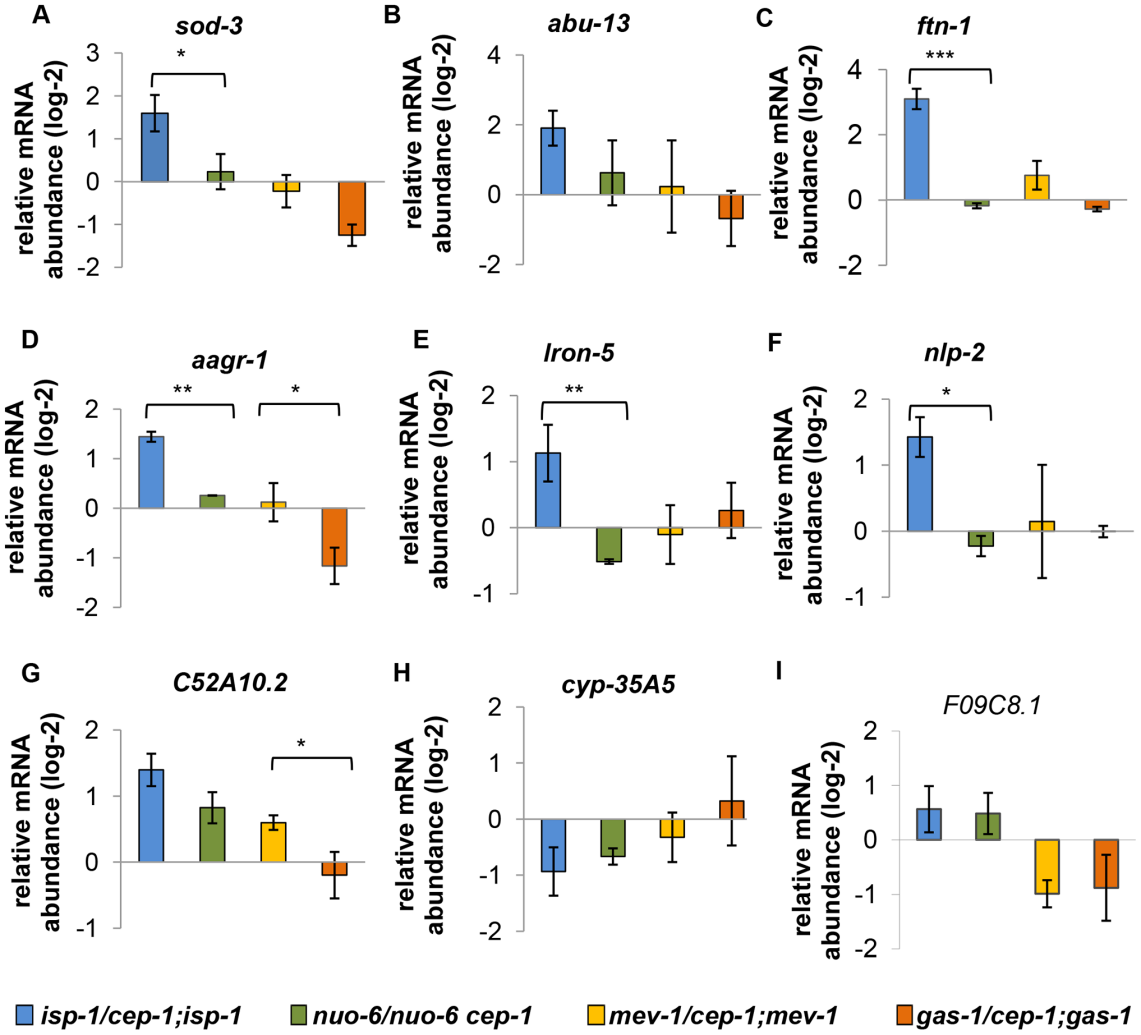
Expression of differentially regulated CEP-1 targets in other ETC mutants. (A–I) These genes are differentially regulated by CEP-1 between *isp-1* and *mev-1* mutants. The relative expression of each gene was normalized to *act-1* and wt. The average log2 ratio between ETC mutants with and without *cep-1* from three independent experiments are plotted. The error bars represent standard errors. Two-tailed t-tests were performed to determine significant differences of the expression of CEP-1 gene targets between the long-lived *isp-1(qm150)* and *nuo-6(qm200)* mutants and the short-lived *mev-1(kn1)* and *gas-1(fc21)* mutants. *p<0.05, ** *p*<0.01, *** *p*<0.001.

The distinct *ftn-1* results led us to consider more broadly whether *isp-1* & *nuo-6* and *gas-1* & *mev-1* share some common molecular signatures, which could account for their similar longevity (extended or shortened, respectively). We examined the expression of a handful of CEP-1-regulated genes that we identified earlier in *isp-1/mev-1* mutants. Using qRT-PCR, we first analyzed the expression of nine genes that were differentially regulated by CEP-1 in *isp-1* and *mev-1* mutants (Fig. 7). Overall, we observed very different patterns for how these genes responded to the absence of *cep-1* in the two long-lived mutants (*isp-1* and *nuo-6*). The majority of genes that showed substantial *cep-1*-dependent induction in *isp-1* mutants barely changed when *cep-1* was depleted in *nuo-6* mutants; only two genes (*cyp-35A5, F09C8.1*) showed consistent, but moderate, *cep-1*-dependent expression changes between *isp-1* and *nuo-6*. Interestingly, the response of these target genes to *cep-1* depletion was more similar between the two short-lived mutants (*mev-1* and *gas-1*). However, it is worth noting that the expression of the majority of these genes barely changed in *mev-1* and *gas-1* mutants in response to *cep-1* deletion (Fig. 7).

**Figure 8:**
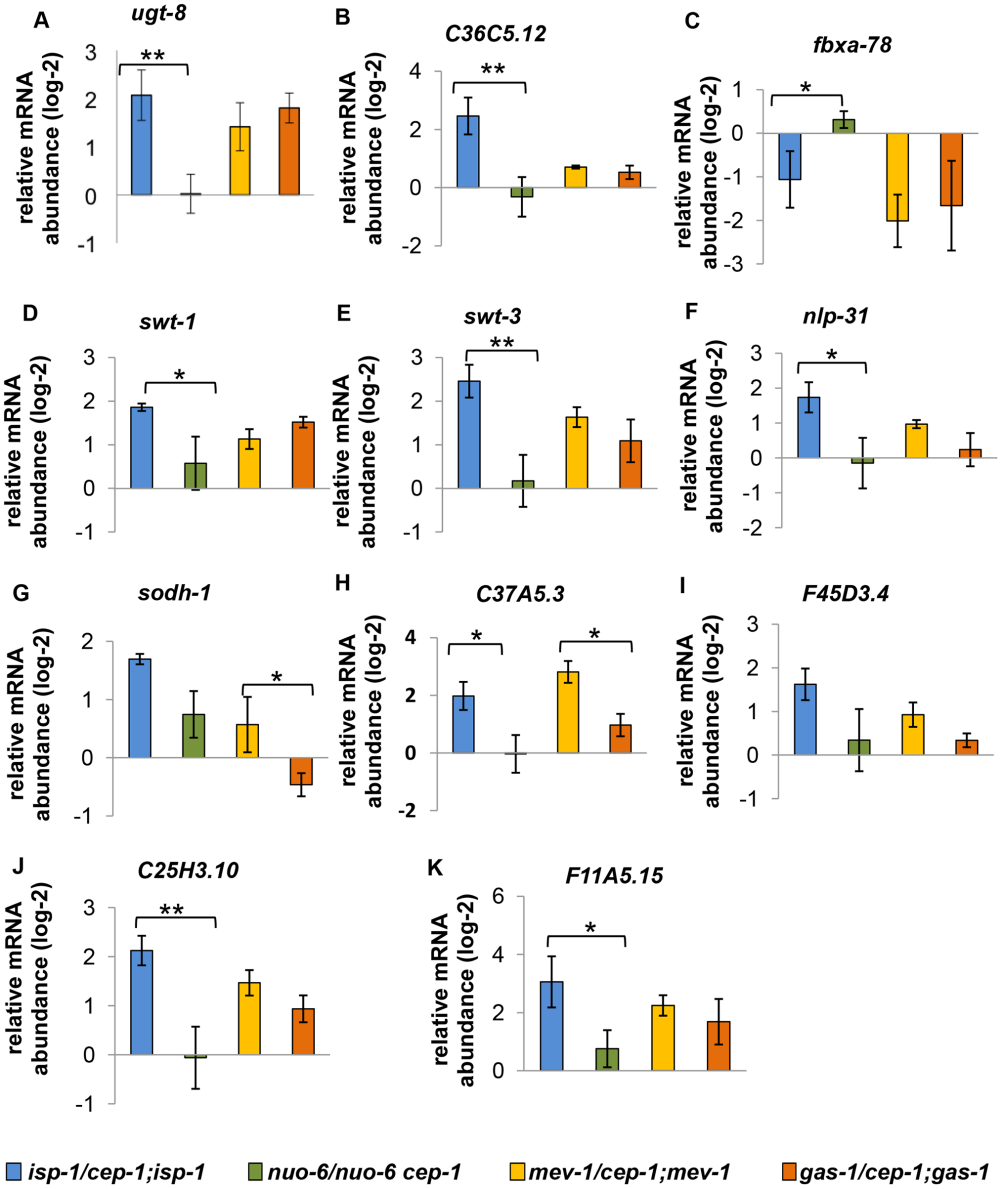
Expression of similarly regulated CEP-1 targets in other ETC mutants. (A–K) These genes are similarly regulated by CEP-1 between *isp-1* and *mev-1* mutants. The relative expression of each gene was normalized to *act-1* and wt. The average log2 ratio between ETC mutants with and without *cep-1* from three independent experiments are plotted. The error bars represent standard errors. Two-tailed t-tests were performed to determine significant differences of the expression of CEP-1 target genes between the long-lived *isp-1(qm150)* and *nuo-6(qm200)* mutants and the short-lived *mev-1(kn1)* and *gas-1(fc21)* mutants. * *p*<0.05, ** *p*<0.01, *** *p*<0.001.

We also assayed the expression of genes that were similarly regulated by CEP-1 in *isp-1* and *mev-1* mutants and observed a similarly discordant pattern (Fig. 8). The regulation of these genes by CEP-1 was strikingly different between *isp-1* and *nuo-6* mutants but was much more similar between *mev-1* and *gas-1* mutants. In the absence of further genome-wide analysis, it is difficult to estimate the extent of common targets that are shared between (the long-lived) *isp-1* and *nuo-6* and between (the short-lived) *mev-1* and *gas-1* mutants. However, based on the small number of genes tested here using qRT-PCR, it appears that CEP-1 regulates some common genes between *mev-1* and *gas-1* mutants, which might account for its ability to restore a normal lifespan in these short-lived mutants. For the long-lived mutants, the situation is more complex; CEP-1 appears to regulate largely distinct genes between *isp-1* and *nuo-6* even though it is required for the extended lifespan of both mutants.

### The CEP-1-regulated transcriptional profiles of mitochondrial dysfunctional mutants are similar to those in worms exposed to UV irradiation

The best-characterized role of mammalian p53 is its ability to respond to DNA damage by inducing cell cycle arrest and repair or apoptosis. We wondered whether the CEP-1-regulated transcriptome changes in mitochondrial mutants would resemble changes observed in response to DNA damage. The global transcriptional profiles of wt or *cep-1* mutant worms treated with UV, gamma, or x-ray irradiation have previously been published [27]–[28]. Using clustering analysis, we compared the CEP-1-dependent global expression profiles in response to mitochondrial dysfunction (in *isp-1* or *mev-1* mutants) to profiles 4 hr after exposure to UV irradiation, 6 hr after gamma-ray, or 2 hr after X-ray (Fig. S6A, Table S8). We observed a higher similarity between the CEP-1-regulated gene expression profiles in response to mitochondrial dysfunction and after UV treatment (correlation coefficient of 0.36) but less after gamma-ray treatment (correlation coefficient of 0.20). The published gene expression profiles after 2 hr of X-ray treatment did not display significant differences from the no treatment control and thus were not included in the downstream analysis.

We analyzed the CEP-1-regulated genes in response to mitochondrial dysfunction and UV and gamma irradiation. We focused on the gene sets that exhibited ≥1.4-fold changes under at least one of the stressed conditions (i.e., *isp-1* mutant, *mev-1* mutant, UV treatment, or gamma irradiation) and performed K-mean clustering to identify the gene sets that were either similarly regulated across all of the stressed conditions or differentially regulated under one or more conditions. The K-mean analysis revealed six distinct clusters (Fig. S6B, Table S9). We employed DAVID to examine the GO terms generated from genes representing each cluster (Fig. S6C, Table S10). Cluster-‘a’ (579 genes) represents genes that were upregulated by CEP-1 in response to all of the stressors. This group is particularly enriched for genes that function in phosphate metabolism, including kinases and phosphatases. Cluster-‘f’ (1,357 genes) represents genes that are also upregulated across all of the stressors but to a lesser extent than in cluster-‘a’. Cluster-‘f’ is enriched for ribosomal and ion transport proteins. Cluster-‘e’ (901 genes) is enriched for growth regulation and represents genes that are repressed by CEP-1 in response to mitochondrial dysfunction and to UV and gamma irradiation. Clusters-‘a’, ‘e’ and ‘f’ together suggest that CEP-1 regulates common transcriptional programs in response to stress, which might in turn induce key signaling pathways to counter the stress and simultaneous suppression of growth. Clusters-‘b’, ‘c’, ‘d’ represent genes that are similarly regulated by CEP-1 in response to mitochondrial dysfunction and UV but are different after gamma irradiation. These gene groups are significantly enriched for nematode cuticle and collagen proteins, cell cycle regulators, glycoproteins and signaling proteins. In summary, both mitochondrial dysfunction and UV irradiation are known to induce ROS, which might reflect the common CEP-1-regulated transcriptional changes observed here.

## Discussion

The major function of p53 is to integrate stress signals and to orchestrate appropriate cellular responses. Under normal, unstressed conditions, p53 is maintained at low levels via proteosome-mediated degradation. Upon stress, p53 levels stabilize and activate stress response programs that range from cell repair to cell death [29]. Our genetic data indicate that mutations in the *C. elegans* ETC subunits *isp-1* and *nuo-6* engage CEP-1 in initiating a stress response that results in a longer lifespan. Conversely, mitochondrial defects, due to mutations in the ETC subunits *mev-1* and *gas-1*, act through CEP-1 to confer a shorter lifespan. Taken together, our data suggest the intriguing possibility that CEP-1 can sense distinct dysfunctional mitochondrial processes and modulate overall longevity accordingly.

How CEP-1 is able to sense mitochondrial dysfunction caused by different ETC mutations remains unclear. ROS have been proposed to be important regulators of p53 [30]. ROS can induce DNA damage, which leads to p53 activation via DNA damage checkpoint pathways. ROS are also known to engage p53 directly through modifying the redox-sensitive Cystein (Cys) residues on p53 [31]. p53 contains several critical Cys residues located within the DNA-binding domain. Importantly, the Cys residues that are required to coordinate zinc and maintain the protein structure that enables interaction with the minor groove of target DNA are conserved between CEP-1 and human p53 [32]. As discussed earlier, many *C. elegans* ETC mutants have been shown to produce elevated levels of mitochondrial ROS. Altered ROS production in the mitochondrial ETC mutants might be coupled to CEP-1 via regulation of the conserved Cys residues. Upon activation, p53 is well-known to regulate the transcription of genes involved in ROS metabolism, including both antioxidant and pro-oxidant genes [33]. Therefore, depending on upstream signaling, CEP-1/p53 can either alleviate ROS stress or promote further ROS accumulation.

Deficiencies in the ETC can also affect cellular energy homeostasis. The *isp-1* mutant has been shown to exhibit higher AMP:ATP ratios compared to wt worms, and *aak-2*, the AMPK α subunit of *C. elegans*, is partially necessary for the extended lifespan of isp-1 mutants [34]. In mammals, the cellular energy sensor AMP kinase (AMPK) is known to directly phosphorylate p53 at Ser15, leading to stabilization and transcriptional activation of p53 [35]. Intriguingly, this Serine residue is conserved between CEP-1 and human p53, and thus, it is possible that an altered AMP:ATP ratio in ETC mutants engages AMPK to regulate CEP-1 function. Further experiments are necessary to definitively identify the signals generated by *C. elegans* ETC mutants that lead to CEP-1 activation.

Although CEP-1 plays opposite roles in *isp-1* and *mev-1* longevity, CEP-1-regulated genes in these two mutants are strikingly similar. This observation suggests that CEP-1 induces similar compensatory responses to restore cellular homeostasis regardless of the specific mitochondrial ETC defect. Our GO analyses suggested, upon mitochondrial dysfunction, that CEP-1 likely promotes a kinases/phosphatase- and/or neuropeptide-mediated signaling cascade, activates metabolic processes poised for defense and detoxification and represses the energy demanding cell cycle program. Although mtUPR has emerged as a key pathway that mediates the physiological outcomes of ETC mutants, we did not observe changes in major mtUPR response genes, such as *atfs-1*, *hsp-6*, or *hsp-60*. Our data suggest that CEP-1 likely does not act through mtUPR to affect the lifespan of ETC mutants.

We were excited to identify a small number of genes that were differentially regulated by CEP-1 in the *isp-1* and *mev-1* mutants, and this gene set was over-represented for genes previously known to modulate aging. We demonstrated that one of these genes, *ftn-1*, was indeed functionally important for the *cep-1*-mediated alterations to longevity in the *isp-1* mutant but not in the *mev-1* mutant, further underscoring CEP-1's functional duality. Ferritin functions to store iron in a non-toxic form, to deposit it in a safe form, and to transport it to areas where it is required. Mitochondria are the sites of iron-sulfur cluster synthesis, which are critical catalytic and structural components of many cellular proteins [36]. Conversely, the presence of iron in mitochondria must be tightly regulated, as free iron can react with ROS and further produce hydroxyl radicals through the Fenton reaction. As *isp-1* encodes an iron-sulphur cluster protein and can bind to iron, iron levels may accumulate in the *isp-1* mutant and thus upregulate *ftn-1* as a compensatory response to restore iron homeostasis. However, this hypothesis requires a thorough investigation of iron homeostasis in *isp-1* and the other ETC mutants to be validated. Additionally, further functional analysis of the small group of genes differentially regulated by CEP-1 in *isp-1* and *mev-1* mutants will likely reveal new genes important for longevity and illuminate how CEP-1 modulates the lifespans of animals with different ETC mutations.

Whereas CEP-1 is essential in mediating the extended lifespan of both *nuo-6* and *isp-1* mutants, our results, albeit based on only a handful of target genes, suggest that CEP-1 regulates distinct genes in each case. This is quite surprising, especially considering that CEP-1 appears to regulate largely similar genes in *gas-1* and *mev-1* mutants. These differences might be due to the specific complex that is compromised and the precise point of electron transport that is defective in the various ETC mutants. During normal electron transport, electrons from complex I or complex II can be passed onto complex III. In the *nuo-6* mutant, where complex I is compromised, fuels can still enter the ETC via complex II, allowing for some degree of electron transport. The situation might be quite different in the *isp-1* mutant, which impairs complex III and would be expected to block electrons transferred from either complex I or complex II. In contrast, a defect in complex I, such as in *gas-1*, or in complex II, such as in *mev-1*, could have a similar consequence on electron transport if fuels are not limiting, as either likely partially compromises electron flow to complex III. Further experiments are necessary to elucidate whether and how different ETC mutations influence CEP-1 activity and gene regulation.

The best known function of CEP-1 in *C. elegans* is its ability to induce apoptosis upon stress. We, however, did not observe expression changes in *egl-1*, the key CEP-1 target for initiating apoptosis [37]. Intriguingly, our results suggest that CEP-1 may protect against physiological apoptosis specifically in the long-lived *isp-1* mutant. This is an aspect of CEP-1 function that remains poorly characterized. Interestingly, analysis of our microarray results revealed that *ced-8*, *ced-9, egl-38*, genes known to regulate apoptosis [37], were specifically regulated in the *isp-1* mutant in a *cep-1*-dependent manner, but their expression was not changed in the *mev-1* mutant. Whether these genes might be central to the ability of CEP-1 to confer protection from physiological apoptosis will warrant further investigation. Future experiments to delineate whether repressed physiological apoptosis is required for the *isp-1* lifespan extension and which CEP-1 target genes might contribute to protection from physiological apoptosis will likely provide important new insights into the function and physiological role of CEP-1/p53.

DNA damage is a major p53-activating stressor, so we compared the CEP-1-mediated transcriptional profiles in mitochondrial mutants and worms treated with UV or gamma irradiation. Our results demonstrated a considerably greater overlap between the CEP-1-regulated transcriptional response induced by ETC disruption and UV irradiation than by gamma irradiation. This might seem surprising given that UV irradiation and gamma irradiation are both genotoxic stressors that cause DNA damage, whereas mitochondrial ETC dysfunction might be considered a metabolic stress. However, UV irradiation is known to produce ROS, which might induce CEP-1 activation in a manner similar to mitochondrial ETC dysfunction, which is also known to induce ROS. Interestingly, the common set of genes that are upregulated by CEP-1 in mitochondrial mutants and in worms exposed to UV and gamma irradiation are highly enriched for kinases and phosphatases but not for DNA damage response genes. Therefore, it is likely that in response to mitochondrial dysfunction and different genotoxic stresses, CEP-1 can induce a core group of signal transduction molecules that initiate a downstream signaling cascade to mount a general stress response. In addition, CEP-1 appears to be able to sense specific damage and induce distinct responses associated with gamma irradiation and UV irradiation or mitochondrial ETC inhibition. Future dissection of the core and damage-specific responses of CEP-1/p53 will enhance our knowledge of the mechanisms that govern the function and regulation of p53, arguably one of the most important and ubiquitous tumor suppressors.

## Materials and Methods

### *C. elegans* strains

All strain stocks were kept at 16°C and grown under standard growth conditions. The following strains were used: Wild-type N2, *isp-1(qm150), nuo-6(qm200), mev-1(kn-1), gas-1(fc21), clk-1(e2519), cep-1(gk138)*, and *Pftn-1::gfp (GA641)*. Standard genetic methods were utilized to construct the following strains: *cep-1(gk138);isp-1(qm150), nuo-6(qm200) cep-1(gk138), cep-1(gk138);clk-1(e2519), cep-1(gk138);mev-1(kn1), cep-1(gk138);gas-1(fc21), Pftn-1::gfp;isp-1(qm150)*, and *Pftn-1::gfp;mev-1(kn-1)*.

### Lifespan analysis

All lifespan assays were performed at 20°C on Nematode Growth Media (NGM) plates seeded with *E. coli* OP50 or RNAi bacteria. A detailed experimental procedure is described in the Supplementary Materials and Methods (Text S1). The survival function of each worm population was estimated using the Kaplan-Meier method, and statistical analysis was performed using a log-rank test (SPSS software). *P*≤0.001 was considered as significantly different from the control population. The independent trials were analyzed separately, and representative experiments are shown in the figures. All of the data from all the trials are shown in Supplementary Table S1. Percent mean lifespan differences from controls were plotted from multiple experiments in Fig. S1, and the data are shown in Supplementary Table S2.

### Apoptosis assay

Worms of each strain were synchronized by picking, and the numbers of apoptotic corpses were counted 48 hours post L4. The corpses were assessed using Differential Interference Contrast (DIC) microscopy under 63× magnification as described in Lant and Derry (2013) [38]. For each strain, at least 3 independent experimental replicates were performed with n≥15, where n = number of gonad arms, for each replicate.

### RNA isolation and microarray preparation

Total RNA was purified from synchronized young adult worms grown at 20°C on OP50 bacteria. Total RNA was isolated using Tri-reagent (Molecular Research Center, Inc.) and purified with the RNeasy kit (Qiagen). cRNA synthesis/amplification, Cy3/Cy5 dye labeling, and hybridization onto Agilent 4×44K *C. elegans* oligonucleotide microarrays were performed as previously described [39]. One of three replicate arrays was dye-flipped.

### Microarray analysis

The normalized expression data were uploaded onto the Princeton University MicroArray database (PUMA [http://puma.princeton.edu]). The raw data were retrieved by SUID (Sequence Unique IDentifier) then averaged by SEQ_NAME with any remaining SUIDs removed. Log2-transformed fold-change data were acquired after setting spot filter criteria, where genes with >80% good data were used. The data were analyzed and visualized using Cluster 3 and TreeView [40]–[41].

The log2 ratios of wt vs. *cep-1(gk138)* with or without UV treatment were obtained from Derry et al. (2007). The log2 ratios of wt vs. *cep-1* after gamma and X-ray irradiation data were obtained from Greiss et al. (2008). For the UV, gamma, and X-ray datasets, we averaged the intensity values of all three wt arrays for each treatment and used it as a reference. Then, we compared the results for each *cep-1* array for the same treatment to the reference to obtain the log2 ratio.

### SAM analysis

SAM analysis [42] was used to identify gene sets that were similarly and differentially regulated in *isp-1* and *mev-1* mutants in a *cep-1*-dependent manner from our microarray data. Log2-transformed fold-change data with no cutoff were submitted to SAM. One class analysis was used to identify genes that similarly changed significantly and consistently in *isp-1* vs. *cep-1;isp-1* and *mev-1* vs. *cep-1;mev-1* datasets. To identify genes differently changed between *isp-1* vs. *cep-1;isp-1* and *mev-1* vs. *cep-1;mev-1* datasets, SAM two-class unpaired analysis with a FDR = 1% was performed. The resulting gene list was compared with the gene list obtained from SAM one-class to exclude any duplicate genes. The unique 71 genes (Table S6) that were present only in SAM two-class analyses were considered differentially regulated between *isp-1* and *mev-1* mutants in a *cep-1*-dependent manner.

### Gene Ontology classification

Worm Base IDs (WBID) of genes identified in SAM and K-mean clusters were input into the Functional annotation-clustering tool in DAVID (http://david.abcc.ncifcrf.gov/) [43] for gene annotation enrichment analysis. Functional annotation clustering was performed with the default criteria, and the enrichment score for each annotation cluster was determined.

### Quantitative Reverse Transcription PCR (qRT-PCR):

Total RNA was isolated from synchronized young adult worms using Tri-reagent (Molecular Research Center, Inc.). cDNAs were synthesized with oligo-dT using the SuperScript III First-Strand Kit (Invitrogen). qRT-PCR reactions were performed using iQ SYBR Green Supermix (BIO-RAD) and the MyiQ Single Color Real-Time PCR Detection System (BIO-RAD). *act-1* was used as the internal control. The qRT-PCR experiments were performed at least in triplicate using independent RNA/cDNA preparations.

### GFP microscopy

For GFP fluorescence images, worms at the L1-L2 stage were paralyzed with levamisole on an agar pad. The GFP signal was visualized at 60× magnification using a Leica DM 5000B microscope. All images were captured with the same intensity and exposure time using Open Lab software.

## Supporting Information

Figure S1 CEP-1 mediated modulation of lifespan during mitochondrial dysfunction. (A) Percent mean lifespan differences between the mutants and wt. Averages of mean lifespans from different experiments are shown. Error bars represent standard deviations (B) Percent mean lifespan differences between the mutants and wt control with or without *cep-1 RNAi* treatment. (C, D) *cep-1 RNAi* treatment suppresses *isp-1* mutant longevity but not *clk-1* mutant longevity.
(TIF)

Figure S2 Cluster analysis of CEP-1-regulated genes in mitochondrial mutants. (A) Hierarchical average linkage gene cluster of CEP-1-regulated genes similarly changed in *isp-1* and *mev-1* mutants. The gene set was identified using SAM one class analysis with FDR = 0.5%. (B) Hierarchical average linkage gene cluster of CEP-1-regulated genes differentially changed in *isp-1* and *mev-1* mutants. The gene set was identified using SAM two class analysis with a FDR = 1%. This list of genes was compared with the gene set that was identified using SAM one class. The 71 genes that were present only in SAM two class analysis were considered CEP-1-regulated genes that differentially changed in *isp-1* and *mev-1* mutants.
(TIF)

Figure S3 Validation of microarray results using qRT-PCR. (A, B) *dct-7* and *fipr-22* represent genes positively regulated by CEP-1 in *isp-1* and *mev-1* mutants. (C, D) *AP-2* and *skr-12* represent genes negatively regulated by CEP-1 in *isp-1* and *mev-1* mutants. (E, F) *sod-3* and *abu-13* represent genes that are differentially regulated by CEP-1 in *isp-1* and *mev-1* mutants. The relative expression of each gene was normalized to *act-1*. The log2 ratios of the average expression for each gene compared to wt from three independent experiments are plotted. Error bars represent standard errors.
(TIF)

Figure S4 Expression analysis of *ftn-1* and *ftn-2* using qRT-PCR. (A) *ftn-1* expression in each mutant strain was normalized to *act-1*. The log2 ratios of the average expression for *ftn-1* compared to wt from three independent experiments are plotted. Error bars represent standard errors. (B) Expression of *ftn-2* in various ETC mutants. The relative expression of each gene was normalized to *act-1*. The average expression ratios for each gene compared to wt from at least two independent experiments are plotted. Error bars represent standard errors.
(TIF)

Figure S5 Percent mean lifespan differences of mutants compared to wt control with or without *ftn-1* and *ftn-2* double RNAi treatment. Averages of mean lifespans from different experiments are shown. Error bars represent standard deviations.
(TIF)

Figure S6 (A) Cluster analysis of the CEP-1-regulated transcriptomes in mitochondrial mutants, and in UV-, gamma- and X-ray-treated animals. Hierarchical single linkage gene cluster was performed and the dendrogram shows the clustered relationship of the arrays. The numbers represent the correlation coefficients of each condition. Each column represents a biological replicate and each row is a gene. (B) K-mean clustering (6 clusters) of CEP-1-regulated genes in *isp-1* and *mev-1* mutants and in UV- and gamma-irradiated animals. Genes that displayed a log2-fold change ≥0.5 in any two individual arrays were selected for clustering. (C) DAVID functional annotation of six K-mean clusters. The numbers represent the enrichment score for each group (score>1.3 is considered as significant).
(TIF)

Table S1 Adult lifespan of all individual experiments.
(XLSX)

Table S2 Percent mean lifespan differences compared to wt.
(XLSX)

Table S3 Rate of development and brood size.
(XLSX)

Table S4 Microarray gene expression (log2) of *isp-1* vs. *cep-1;isp-1* and *mev-1* vs. *cep-1;mev-1*.
(XLSX)

Table S5 Similarly expressed CEP-1-regulated genes between *isp-1* and *mev-1* mutants identified by SAM one-class analysis.
(XLSX)

Table S6 CEP-1-regulated genes that are differentially changed in the *isp-1* and *mev-1* mutants.
(XLSX)

Table S7 Functional annotation of different sets of CEP-1-regulated genes in *isp-1* and *mev-1* mutants by DAVID.
(XLSX)

Table S8 CEP-1-regulated transcriptomes in mitochondrial mutants and in UV-, gamma- and X-ray-irradiated animals.
(XLSX)

Table S9 Genes that changed ≥1.4-fold under at least one of the stressed conditions (*isp-1* mutant, *mev-1* mutant, UV treatment, or gamma irradiation).
(XLSX)

Table S10 K-mean clustering identifies gene groups that were up or down regulated under at least one of the stressed conditions.
(XLSX)

Text S1 Supplementary Materials and Methods.
(PDF)
