# Supplementary figures and images for: CEP-1, the Caenorhabditis elegans p53 Homolog, Mediates Opposing Longevity Outcomes in Mitochondrial Electron Transport Chain Mutants
Source: PLoS Genet. 2014 Feb 27;10(2):e1004097. doi: 10.1371/journal.pgen.1004097 (PMC3937132; doi:10.1371/journal.pgen.1004097)

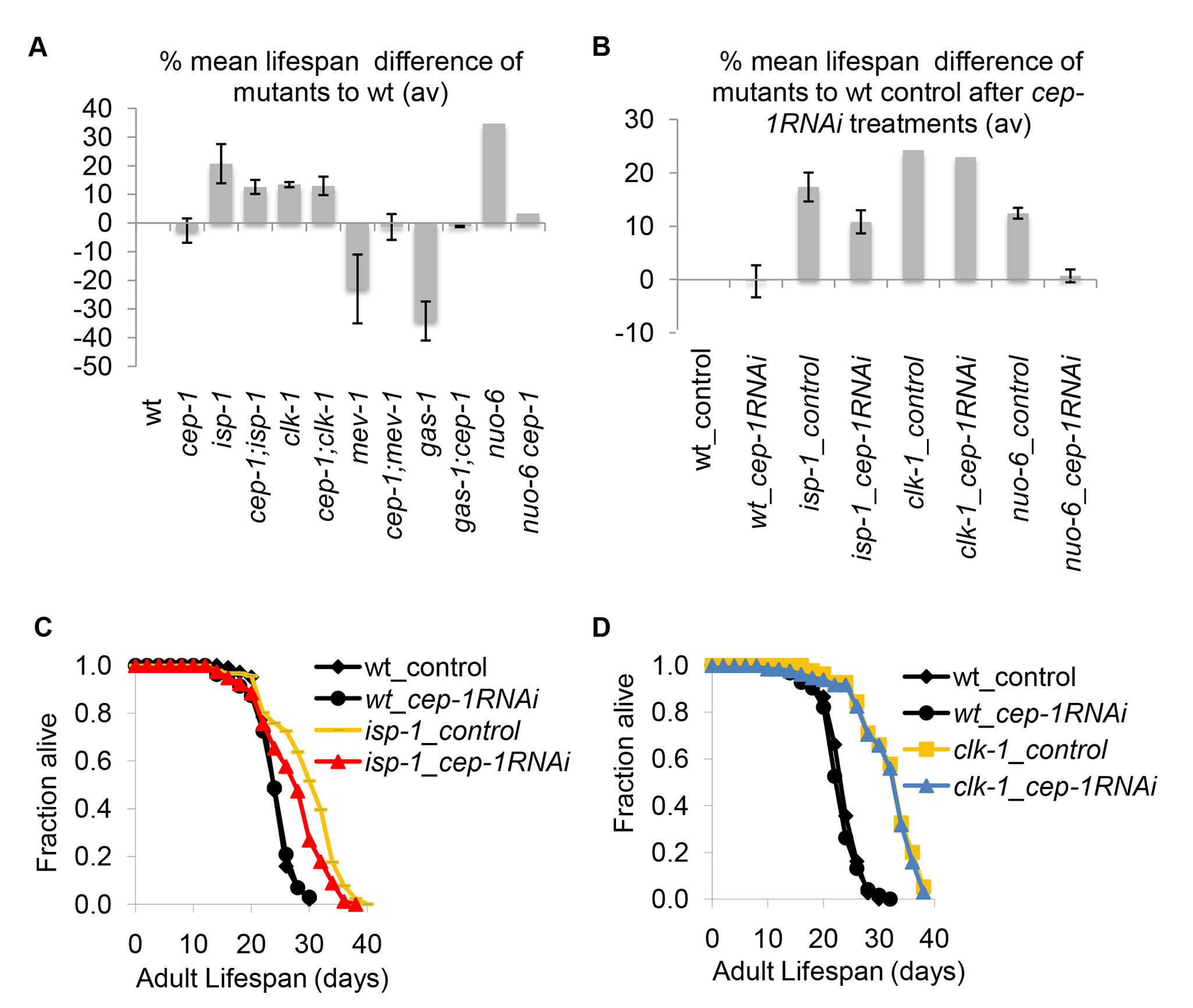

Supplement: Figure S1 — CEP-1 mediated modulation of lifespan during mitochondrial dysfunction. (A) Percent mean lifespan differences between the mutants and wt. Averages of mean lifespans from different experiments are shown. Error bars represent standard deviations (B) Percent mean lifespan differences between the mutants and wt control with or without cep-1 RNAi treatment. (C, D) cep-1 RNAi treatment suppresses isp-1 mutant longevity but not clk-1 mutant longevity. (TIF) [file pgen.1004097.s001.tif]

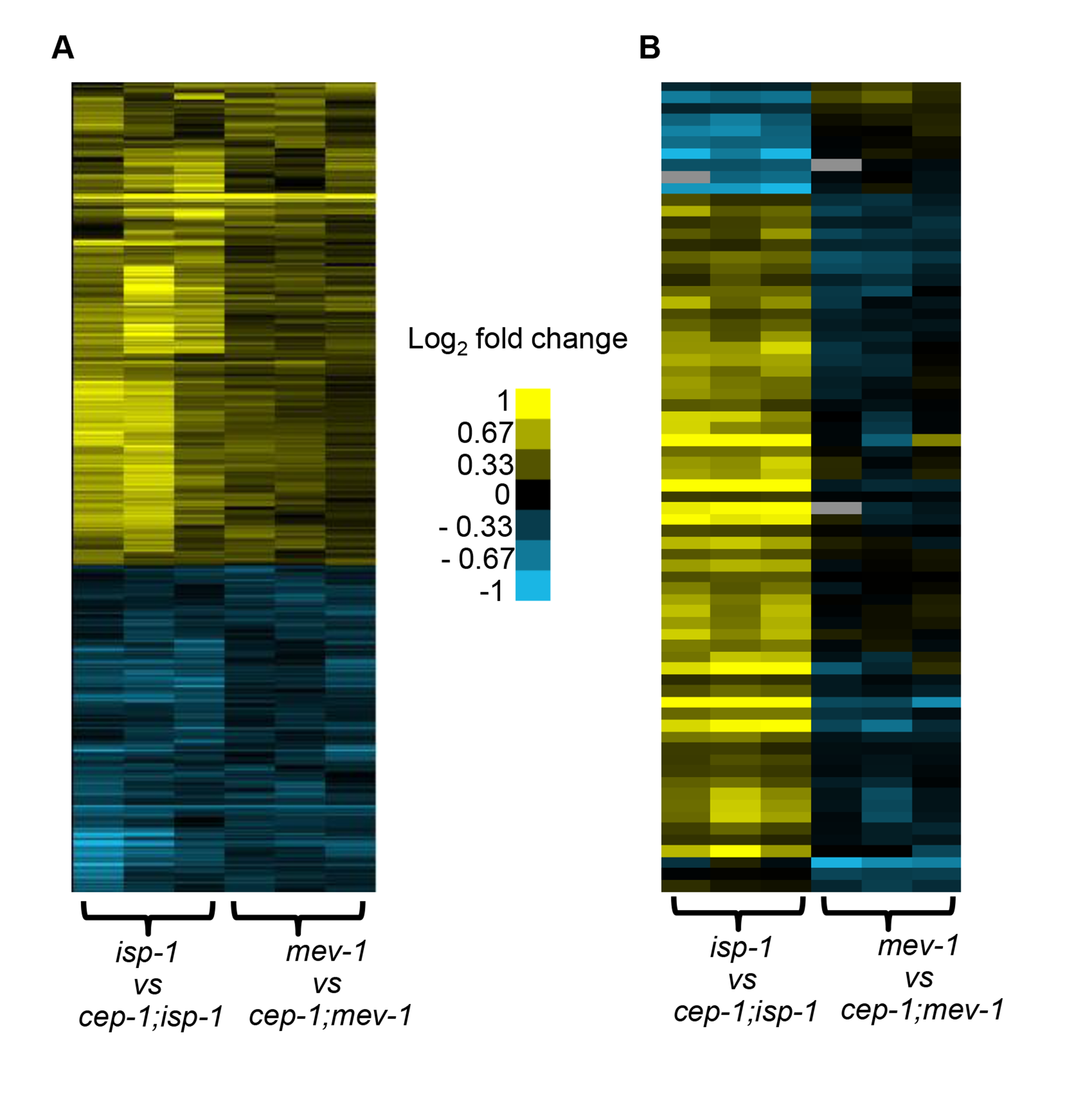

Supplement: Figure S2 — Cluster analysis of CEP-1-regulated genes in mitochondrial mutants. (A) Hierarchical average linkage gene cluster of CEP-1-regulated genes similarly changed in isp-1 and mev-1 mutants. The gene set was identified using SAM one class analysis with FDR = 0.5%. (B) Hierarchical average linkage gene cluster of CEP-1-regulated genes differentially changed in isp-1 and mev-1 mutants. The gene set was identified using SAM two class analysis with a FDR = 1%. This list of genes was compared with the gene set that was identified using SAM one class. The 71 genes that were present only in SAM two class analysis were considered CEP-1-regulated genes that differentially changed in isp-1 and mev-1 mutants. (TIF) [file pgen.1004097.s002.tif]

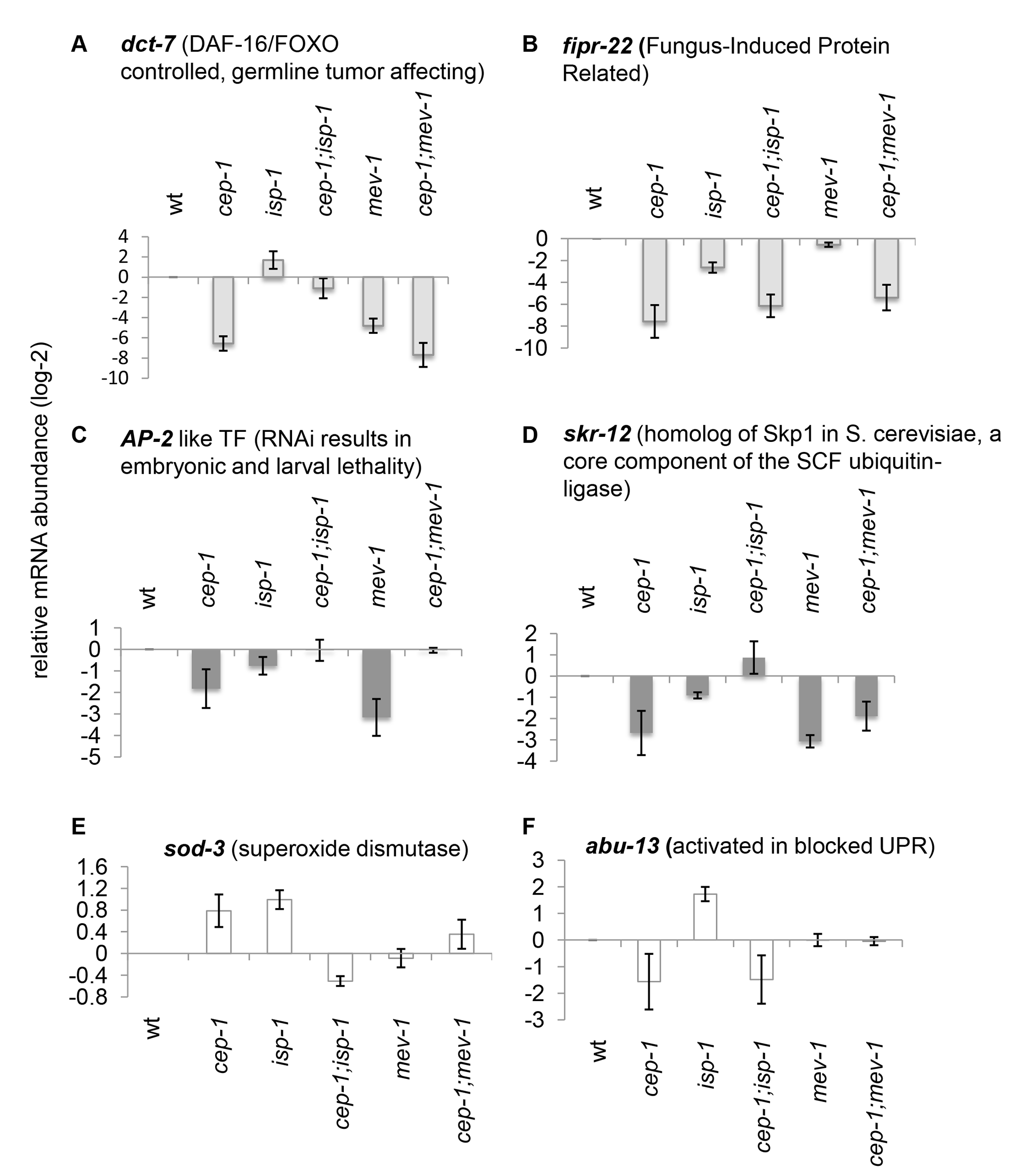

Supplement: Figure S3 — Validation of microarray results using qRT-PCR. (A, B) dct-7 and fipr-22 represent genes positively regulated by CEP-1 in isp-1 and mev-1 mutants. (C, D) AP-2 and skr-12 represent genes negatively regulated by CEP-1 in isp-1 and mev-1 mutants. (E, F) sod-3 and abu-13 represent genes that are differentially regulated by CEP-1 in isp-1 and mev-1 mutants. The relative expression of each gene was normalized to act-1. The log2 ratios of the average expression for each gene compared to wt from three independent experiments are plotted. Error bars represent standard errors. (TIF) [file pgen.1004097.s003.tif]

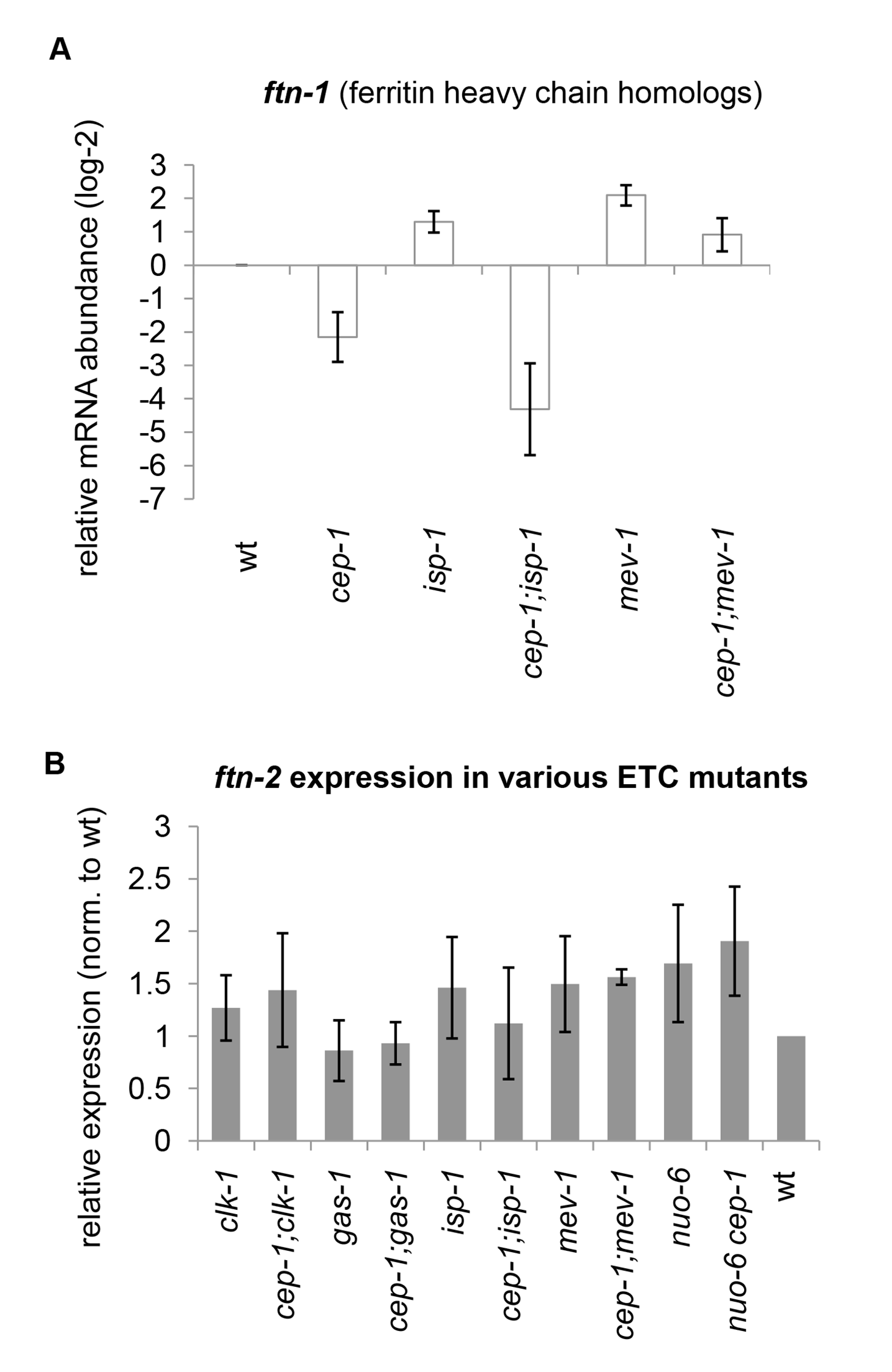

Supplement: Figure S4 — Expression analysis of ftn-1 and ftn-2 using qRT-PCR. (A) ftn-1 expression in each mutant strain was normalized to act-1. The log2 ratios of the average expression for ftn-1 compared to wt from three independent experiments are plotted. Error bars represent standard errors. (B) Expression of ftn-2 in various ETC mutants. The relative expression of each gene was normalized to act-1. The average expression ratios for each gene compared to wt from at least two independent experiments are plotted. Error bars represent standard errors. (TIF) [file pgen.1004097.s004.tif]

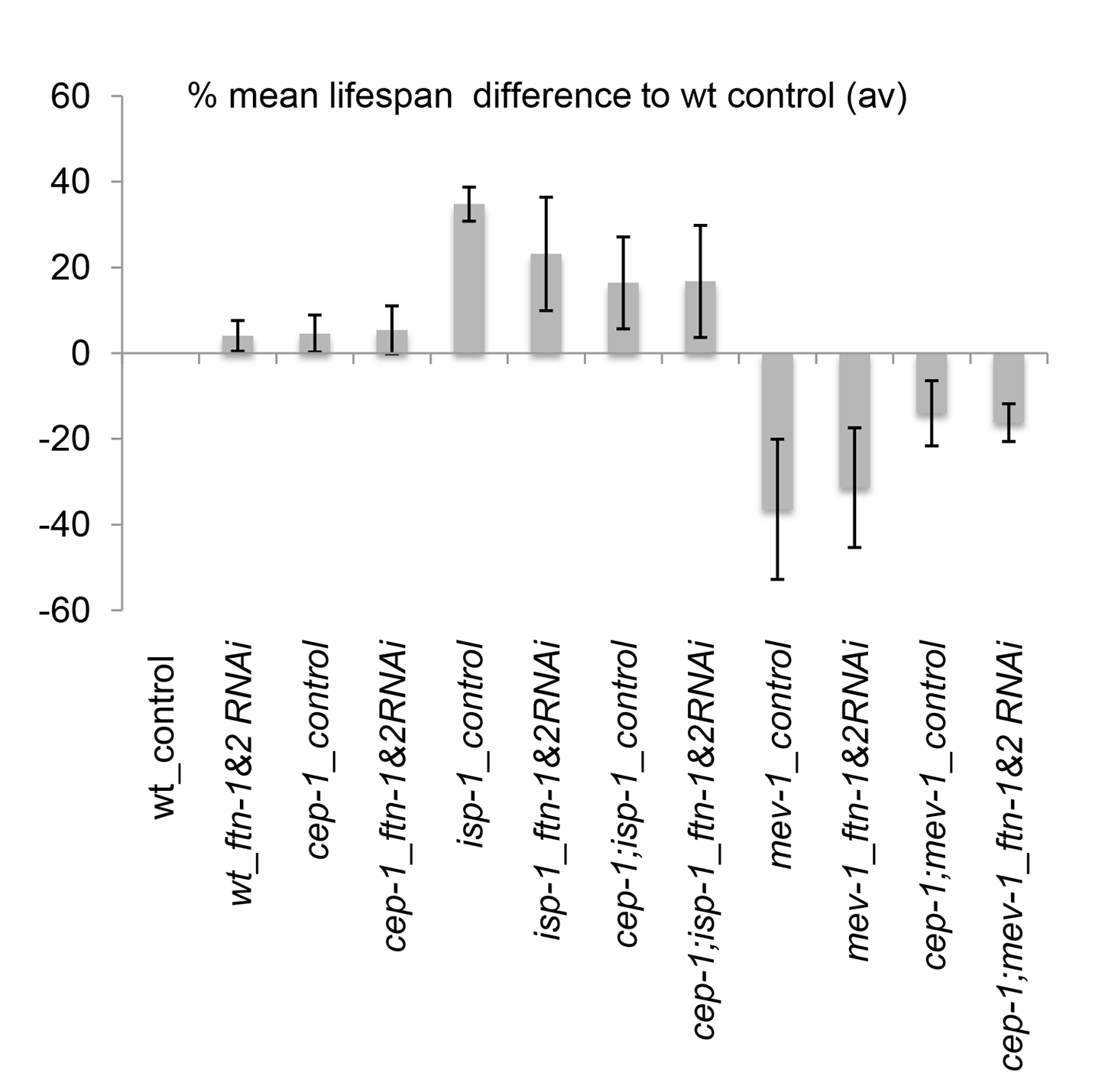

Supplement: Figure S5 — Percent mean lifespan differences of mutants compared to wt control with or without ftn-1 and ftn-2 double RNAi treatment. Averages of mean lifespans from different experiments are shown. Error bars represent standard deviations. (TIF) [file pgen.1004097.s005.tif]

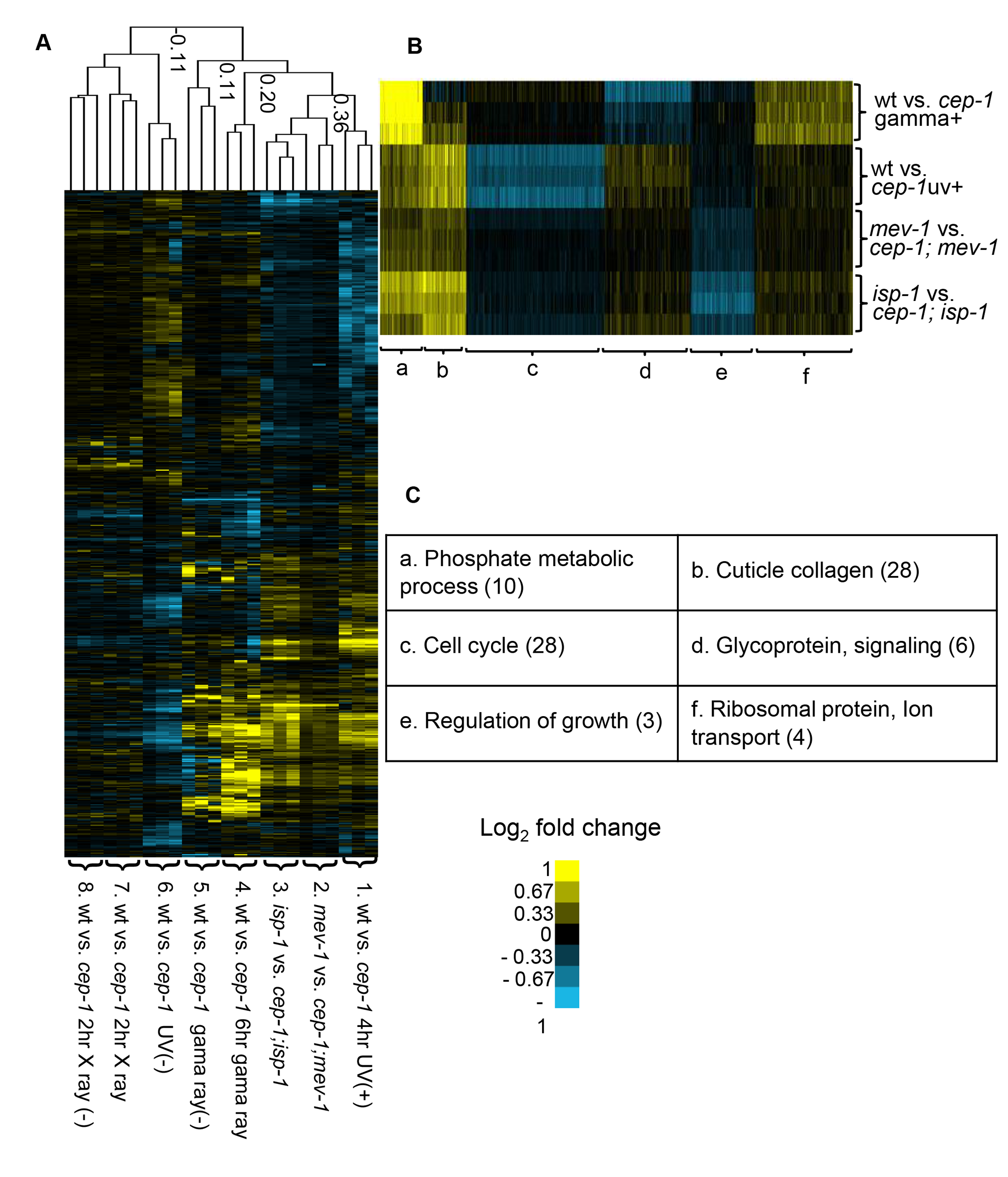

Supplement: Figure S6 — (A) Cluster analysis of the CEP-1-regulated transcriptomes in mitochondrial mutants, and in UV-, gamma- and X-ray-treated animals. Hierarchical single linkage gene cluster was performed and the dendrogram shows the clustered relationship of the arrays. The numbers represent the correlation coefficients of each condition. Each column represents a biological replicate and each row is a gene. (B) K-mean clustering (6 clusters) of CEP-1-regulated genes in isp-1 and mev-1 mutants and in UV- and gamma-irradiated animals. Genes that displayed a log2-fold change ≥0.5 in any two individual arrays were selected for clustering. (C) DAVID functional annotation of six K-mean clusters. The numbers represent the enrichment score for each group (score>1.3 is considered as significant). (TIF) [file pgen.1004097.s006.tif]
