# Supplementary material for: CEP-1, the Caenorhabditis elegans p53 Homolog, Mediates Opposing Longevity Outcomes in Mitochondrial Electron Transport Chain Mutants
Source: PLoS Genet. 2014 Feb 27;10(2):e1004097. doi: 10.1371/journal.pgen.1004097 (PMC3937132; doi:10.1371/journal.pgen.1004097)
Supplement: Text S1 — Supplementary Materials and Methods. (PDF) [file pgen.1004097.s017.pdf]

Supplementary Materials and Methods:

Lifespan analysis:

For lifespan experiments using OP50, bacteria were grown overnight at 37°C in LB with streptomycin. The OD at 600 nm of the bacterial cultures was adjusted to the same value (1x bacteria: values at 0.175 for 1/10 dilution). The 35mm NGM plates were seeded with 150μL 5X OP50 bacterial concentration. Plates that would be used for transferring worms during lifespan assays were prepared by adding FUDR to prevent progeny, to a final concentration of 50 μg/ml per plate, except for lifespan experiments for *gas-1* mutant worms where only 5 μg/ml FUDR concentration was used. For RNAi experiments, HT115 bacteria containing vectors expressing dsRNA were grown at 37°C in LB with 50μg/mL ampicillin and 15μg/mL tetracycline to OD 0.8. 150μL of 5X bacterial culture were plated to 35mm NGM plates containing 4mM IPTG and induced overnight at room temperature. Well-fed gravid adult worms were allowed to lay eggs and progeny were grown at 20°C until young adult stage. The synchronized adults were transferred to fresh FUDR-containing plates at day 0, 2, 4, 6 and 8 of adulthood. The worms were incubated at 25°C for the first three days of adulthood to reduce vulva protrusion defects. The adult worms were scored every other day and worms that did not move when gently prodded by a platinum wire pick were recorded as dead. Worms that bagged, crawled onto the wall of the plate, or had a large protruding vulva were censored on the day of the event.

Developmental timing and brood size assay:

Developmental timing of mutant worms compared to wt were determined by placing 3 to 5 gravid adult worms on OP50 plates, and the animals were allowed to lay eggs at 20°C. After 6 hr, adult worms were removed from the plates, which were kept at 20°C. The developmental stages of wt and mutants worms were assayed daily using a dissecting microscope. At 60 hr-post egg lay, all wt worms reached adulthood (100%). Approximately 40 worms of each mutant genotype were mounted on agar pads and observed under a compound microscope. The developmental stage of worms was noted, quantified, and the average percentage was plotted in Fig. 1C (Table S3A).

To determine brood size, 5 to 10 young adults were singled onto fresh plates incubated at 20°C and transferred onto fresh plates every 12 hr to prevent overcrowding until egg laying ceased. The progeny production on each plate was counted 36 hr after removal of the parent. The mean self-brood size obtained for each strain was compared using Student's *t* test (Fig 1D, Table S3B).

Microarray analysis:

Hybridized microarray slides were washed according to Agilent instructions, and scanned immediately on the Agilent DNA microarray scanner (G2505B) using one color scan setting for 4x44k array slides (Scan Area 61x21.6 mm, Scan resolution 5um, Dye channel is set to Red & Green and both the Red and Green PMT is set to 100%). The scanned images were analyzed with Feature Extraction Software 9.1 (Agilent) using parameters (protocol GE1-105_DEC8 and Grid: 012391_D_20060331) to obtain background subtracted and spatially detrended Processed Signal intensities. Features flagged in Feature Extraction as Feature Non-uniform outliers were excluded.
